# Influence of the Surface Chemistry of Metal–Organic Polyhedra in Their Assembly into Ultrathin Films for Gas Separation

**DOI:** 10.1021/acsami.2c06123

**Published:** 2022-06-03

**Authors:** Inés Tejedor, Miguel A. Andrés, Arnau Carné-Sánchez, Mónica Arjona, Marta Pérez-Miana, Javier Sánchez-Laínez, Joaquín Coronas, Philippe Fontaine, Michel Goldmann, Olivier Roubeau, Daniel Maspoch, Ignacio Gascón

**Affiliations:** † Instituto de Nanociencia y Materiales de Aragón (INMA), CSIC and Universidad de Zaragoza, Zaragoza 50009, Spain; ‡ Departamento de Química Física, Universidad de Zaragoza, Zaragoza 50009, Spain; § Catalan Institute of Nanoscience and Nanotechnology (ICN2), CSIC and The Barcelona Institute of Science and Technology, Campus UAB, Bellaterra, Barcelona 08193, Spain; ∥ Chemical and Environmental Engineering Department, Universidad de Zaragoza, Zaragoza 50018, Spain; ⊥ Synchrotron SOLEIL, L’Orme des Merisiers, Saint-Aubin, BP 48, Gif-sur-Yvette 91192, France; # Institut des NanoSciences de Paris, UMR 7588 CNRS, Sorbonne Université, 4 Place Jussieu, Paris Cedex 05 75252, France

**Keywords:** metal−organic polyhedra (MOP), ultrathin films, surface functionalization, CO_2_ separation, MOP assembly

## Abstract

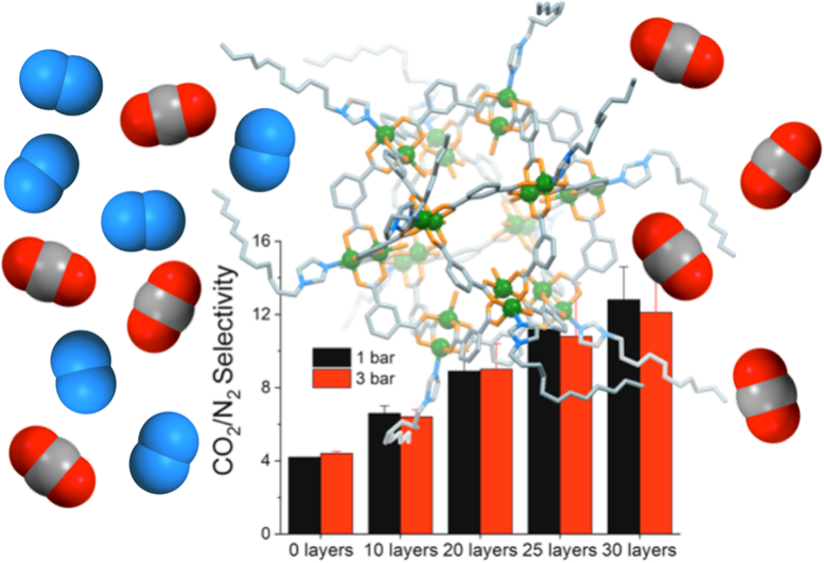

The formation of ultrathin films of Rh-based porous metal–organic polyhedra (Rh-MOPs) by the Langmuir–Blodgett method has been explored. Homogeneous and dense monolayer films were formed at the air–water interface either using two different coordinatively alkyl-functionalized Rh-MOPs (HRhMOP(diz)_12_ and HRhMOP(oiz)_12_) or by *in situ* incorporation of aliphatic chains to the axial sites of dirhodium paddlewheels of another Rh-MOP (OHRhMOP) at the air–liquid interface. All these Rh-MOP monolayers were successively deposited onto different substrates in order to obtain multilayer films with controllable thicknesses. Aliphatic chains were partially removed from HRhMOP(diz)_12_ films post-synthetically by a simple acid treatment, resulting in a relevant modification of the film hydrophobicity. Moreover, the CO_2_/N_2_ separation performance of Rh-MOP-supported membranes was also evaluated, proving that they can be used as selective layers for efficient CO_2_ separation.

## 1 Introduction

Intrinsically porous metal–organic polyhedra^1^ (MOPs) are a class of metal–organic cages^2^ obtained from the self-assembly of metal ions and organic linkers. Compared to metal–organic frameworks,^3^ widely used for different applications that make use of their permanent porosity (separations,^4^ sensing,^5^ and drug delivery,^6^ among others), MOPs present some relevant advantages due to their molecular nature, such as tailorable solubility and easy processability.^7^ These properties make MOPs excellent candidates for molecular separation processes in liquid phases, including cargo transfer between immiscible solvents,^8^ the formation of ion channels,^9^ and the development of membranes for gas separation.^10^

Previous studies have shown that cuboctahedral rhodium-based MOPs (Rh-MOPs), which are assembled from 1,3-benzenedicarboxylate and dirhodium paddlewheels,^11^ offer very interesting possibilities for separation processes. The cavity of Rh-MOPs can be used for selective entrapment of gases/molecules,^12^ whereas their reactive surface can be used to coordinatively attach selected molecules.^13^ The chemical robustness of Rh-MOPs enables them to sustain their performance even when exposed to harsh conditions, such as exposure to water, acids, or competing coordinating ligands.^14^ In addition, the surface of Rh-MOPs can be functionalized to modulate their solubility from water to polar/non-polar organic solvents, which increases their processability and enables using them under homogeneous conditions or shaping them into functional macroscopic objects. In such a context, we^15^ and Yin and co-workers^16^ recently demonstrated that alkyl-functionalized MOPs can form stable monolayers at the air–liquid interface. The self-assembly of these MOP films can be studied *in situ* during film formation at different surface densities (obtained from surface pressure–area isotherms) using specific characterization techniques such as Brewster angle microscopy (BAM) or neutron reflectivity (NR), which provide relevant information about the organization and structure of the films. Moreover, these films can be sequentially transferred onto appropriate solid substrates by horizontal (Langmuir–Schaefer, LS) or vertical (Langmuir–Blodgett, LB) depositions to obtain multilayer films with the required thickness.

Dense and ultrathin MOP films would be the basis for the development of efficient MOP-based membranes for separation technologies. For instance, LS multilayer films (*ca.* 60 nm) fabricated with the cuboctahedral Rh-MOP named C_12_RhMOP, with the formula [Rh_2_(C_12_-bdc)_2_]_12_ (where C_12_-bdc is 5-dodecyloxybenzene-1,3-dicarboxylate), showed remarkable CO_2_ permeance and CO_2_/N_2_ selectivity values in post-combustion.^15^

In this study, we expand the fabrication of Rh-MOP films to different compounds with aliphatic chains coordinated to Rh-MOP axial sites. These chains can be incorporated before spreading Rh-MOP onto the liquid surface or *in situ* at the air–liquid interface. Moreover, an acid treatment after film formation allows a partial ligand cleavage, which modifies the hydrophobicity of the film surface, opening the possibility to post-synthetically modify the properties of ultrathin MOP-based films.

## 2 Materials and Methods

### 2.1 Synthesis and Post-Synthetic Modification of Rh-MOPs

Both ligands 1-dodecyl-1*H*-imidazole (diz) and 1-octyl-1*H*-imidazole (oiz) were synthesized according to a reported procedure.^17^ OHRhMOP (formula: [Rh_2_(OH-BDC)_2_]_12_, where OH-BDC is 5-hydroxy-1,3-benzenedicarboxylate) was synthesized according to the reported procedure.^14^ HRhMOP(diz)_12_ (formula: [Rh_2_(BDC)_2_(diz)_1_]_12_, where BDC is 1,3-benzenedicarboxylate) was synthesized according to the reported procedure.^7b^ HRhMOP(oiz)_12_ (formula: [Rh_2_(BDC)_2_(oiz)_1_]_12_) was synthesized following a procedure similar to HRhMOP(diz)_12_. To this end, 100 mg of HRhMOP (formula: [Rh_2_(BDC)_2_]_12_) was dispersed in 20 mL of dichloromethane (DCM). Then, 42.05 mg of oiz (15 equiv) was added. The solution was sonicated for 5 min and filtered. The filtrate was evaporated under vacuum. The solid residue was washed twice with ether to remove unreacted oiz. Finally, the obtained purple solid was dried under vacuum.

OHRhMOP was alkyl-functionalized in solution *via* coordination chemistry with an excess (1:25) of the N-donor ligand (diz) to obtain OHRhMOP(diz)_12_ (formula: [Rh_2_(OH-BDC)_2_(diz)_1_]_12_). The same ligand was also used to study the *in situ* functionalization of OHRhMOP at the air–liquid interface. The bulk synthesis was performed by adding 0.9 μL of diz (*ca.* 3.8 × 10^–3^ mmol) to a dispersion of 1 mg of OHRhMOP (*ca.* 1.5 × 10^–4^ mmol) in 2 mL of THF. The insoluble green powder of OHRhMOP was immediately dissolved after the addition of diz. The resultant purple solution presented its maximum absorption in the visible region at *ca.* 552 nm (Figure S1), which indicates that all the dirhodium paddlewheels in the Rh-MOP structure are coordinated to one diz, as it has been previously reported.^14^ OHRhMOP dissolved in a 1:5 methanol/chloroform mixture (the same mixture used for the preparation of Langmuir films) showed its maximum absorbance at 588 nm (also plotted in Figure S1 for comparison).

### 2.2 Langmuir and LS Film Fabrication and Characterization

Diluted solutions (*ca.* 3.0 × 10^–6^ M) of C_12_RhMOP, HRhMOP(diz)_12_, and HRhMOP(oiz)_12_ were prepared in DCM, while OHRhMOP was dissolved in a mixture of 1:5 methanol/chloroform (*ca.* 6.0 × 10^–5^ M). Appropriate volumes of these solutions (*ca.* 1000–1200 μL for C_12_RhMOP, HRhMOP(diz)_12_, and HRhMOP(oiz)_12_ and 5000 μL for OHRhMOP) were spread on the water subphase for the formation of Rh-MOP films.

For the interfacial formation of alkyl-functionalized Rh-MOPs at the air–water interface, 3150 μL of OHRhMOP solution and an appropriate volume of diz solution (concentration of 2.25 × 10^–4^ M in DCM), in order to achieve the desired molar proportion Rh-MOP/ligand, were spread successively onto the air/water interface.

In all experiments, after solvent evaporation (*ca.* 15 min), the floating molecules were compressed to induce their assembly into an ordered film.

A NIMA 702BAM Langmuir trough (720 × 100 mm) equipped with two mobile symmetrical barriers was used to obtain surface pressure versus area (π–*A*) isotherms and BAM images. Each π–*A* isotherm was registered at least three times in order to check the reproducibility of the results before further experiments (BAM image acquisition, film transfer onto different substrates, *etc.*) were carried out. BAM images were acquired using a KSV NIMA Micro BAM equipped with a red laser (659 nm, 50 mW) as the light source. The incidence angle was fixed at 53.1°, and a black quartz plate was placed inside the trough as a light trap. The optics of the system provided a spatial resolution of 6 μm per pixel in the water surface plane and a field of view of 3600 × 4000 μm^2^.

LS films were fabricated using a KSV-NIMA model KN 2003 (580 × 145 mm), also arranged in a symmetrical double-barrier configuration.

Both troughs were placed inside closed cabinets in a dedicated facility to limit the presence of dust and at a constant temperature (20 ± 1 °C). Ultra-pure Milli-Q water (resistivity 18.2 MΩ·cm) was used in all the experiments as the subphase. The films were compressed in all cases at a constant speed of 8 cm^2^/min, and surface pressure was continuously monitored during the experiments by means of a Wilhelmy balance using a filter paper plate.

LS films were transferred after film compression and stabilization of the desired surface pressure, keeping the substrates parallel to the water surface using a vacuum pump-based horizontal dipping clamp (KSV KN-006). The substrate was approached to the water surface at a vertical speed of 1 mm/min. Once the substrate touched the film, it was withdrawn at a vertical speed of 10 mm/min. After each transfer, films were dried with N_2_ at ambient temperature, and the transferences were repeated, when required, as many times as necessary to obtain films with the desired number of Rh-MOP layers.

The UV–vis spectra of the Rh-MOP solutions and films deposited onto the quartz substrates were recorded on a Varian Cary 50 Bio spectrophotometer.

LS films were characterized through atomic force microscopy (AFM) under ambient conditions using either a NTEGRA Aura microscope from NT-MDT (semicontact mode employing a SF005&AU006NTF head) or a Bruker Multimode 8 microscope, which is equipped with a NanoScope V control unit (tapping mode). AFM data were collected using silicon tips with typical spring constants and resonant frequencies of 3.5–5 N·m^–1^ and 90–210 kHz, respectively.

The Rh-MOP mass deposited onto QCM substrates (5 MHz AT-cut QCM crystals from Stanford Research Systems) was determined using a Stanford Research Systems (SRS) QCM200 system equipped with a QCM25 crystal oscillator working at 5 MHz. QCM crystals were placed in a O100RH Kynar crystal holder before and after film transfer, and the mass increases were calculated from frequency changes using the Sauerbrey equation, Δ*f* = −*C*_f_Δ*m*, where Δ*f* is the observed frequency change in Hz, *C*_f_ is the sensitivity factor of the QCM crystal provided by the manufacturer (0.0566 Hz·cm^2^/ng), and Δ*m* is the change in mass per unit area.

X-ray photoelectron spectroscopy (XPS) spectra were acquired on a Kratos AXIS ultra DLD spectrometer with a monochromatic Al Kα X-ray source (1486.6 eV) using a pass energy of 20 eV. The photoelectron take-off angle was 90° with respect to the sample plane. The XPS binding energies reported in this work were referenced to the C 1s peak at 284.6 eV. The data treatment was done using CasaXPS software. The Rh 3d high-resolution spectra were simulated using Shirley-type background and the components detailed in Table S1.

### 2.3 Synchrotron Characterization of Langmuir Films

*In situ* grazing-incidence X-ray diffraction (GIXD) and grazing-incidence small-angle X-ray scattering (GISAXS) measurements at the air–water interface were performed *in situ* at SIRIUS Beamline of Synchrotron SOLEIL (Saint Aubin, France).^18^ The measurements were performed using an incident X-ray beam of 8 keV (λ = 0.155 nm), a beam size of 0.1 × 0.5 mm^2^ (*V* × *H*) for GISAXS and 0.1 × 2.0 mm^2^ (*V* × *H*) for GIXD, and an incidence angle of 2.0 mrad with the water surface below the total external reflection critical angle value of the air–water interface (2.7 mrad at 8 keV). GISAXS measurements were done using a 2D PILATUS3 1M (Dectris, Switzerland) detector located at 4.5 m from the sample. A vertical tungsten rod was used as a beam-stop for the direct and reflected beams. A flight path tube flushed with helium gas was located between the sample and the detector in order to reduce absorption and scattering by air. The GIXD setup used a 2D PILATUS2 (Dectris, Switzerland) detector combined with a Soller collimator of 0.05° resolution. This detection setup was continuously scanned over the in-plane 2θ angle in order to record the horizontal and vertical distribution intensity. Peak adjustment was performed with the *Q*_*z*_-integrated intensity.

Monolayers were prepared in a dedicated Langmuir trough^18^ enclosed in a gastight chamber flushed by water-saturated helium gas flow to reduce gas scattering and to avoid the damage of the monolayer by the beam. The temperature was kept constant thanks to a water circulating bath at 20 ± 1 °C.

### 2.4 Rh-MOP Films for Gas Separation Studies

For gas separation studies, Rh-MOP LS films were deposited onto permeable poly[1-(trimethylsilyl)-1-propyne] (PTMSP) supports of a thickness of *ca.* 80 μm. Scheme S1 illustrates the LS sequential deposition of MOP films onto the PTMSP membrane in order to achieve the desired number of MOP monolayers in the selective layer. PTMSP supports were prepared following a procedure previously described.^19^ PTMSP (Cymit Quimica, >95%) was first dissolved in toluene (analytical reagent purchased from VWR Chemicals, >99.5%) at room temperature (solution concentration 1.9 wt %). Then, the solution was poured on a glass Petri dish and allowed to dry for 72 h at room temperature. The obtained films were immersed in methanol (Sigma-Aldrich, 99.8%) for 24 h to remove the traces of toluene. Before using them, the PTMSP supports were gently dried with paper sheets.

Rh-MOP/PTMSP membranes were cut in circular areas of 2.12 cm^2^ for conducting gas separation studies at 35 °C and two different feed pressures (1 and 3 bar). The membranes were assembled into a module consisting of two stainless steel pieces and a 316LSS macroporous disk support (from Mott Co.) with a 20 μm nominal pore size, gripped inside with Viton O-rings. The permeation module was placed in a UNE 200 Memmert oven to control the temperature of the module. Gas separation measurements were carried out by feeding a 10/90 in volume CO_2_/N_2_ mixture (100 cm^3^(STP)/min) to the feed side by means of two mass flow controllers (Alicat Scientific, MC-100CCM-D), while the permeate side of the membrane was swept with a 4.5 cm^3^(STP)/min mass flow-controlled stream of He at 1 bar (Alicat Scientific, MC-5CCM-D). The permeate concentrations of CO_2_ and N_2_ were analyzed online by an Agilent 3000 A micro-gas chromatograph equipped with a thermal conductivity detector . Permeance was calculated in GPU [10^–6^ cm^3^(STP)/(cm^2^ s cmHg)] once the steady state was reached (after about 3 h). The separation selectivity was obtained as the ratio of CO_2_ and N_2_ permeances. At least two membrane samples of each type were fabricated and measured to provide the corresponding error estimations.

## 3 Results and Discussion

### 3.1 Assembly of Soluble Rh-MOPs into Thin Films at the Air–Liquid Interface

**Figure 1:**
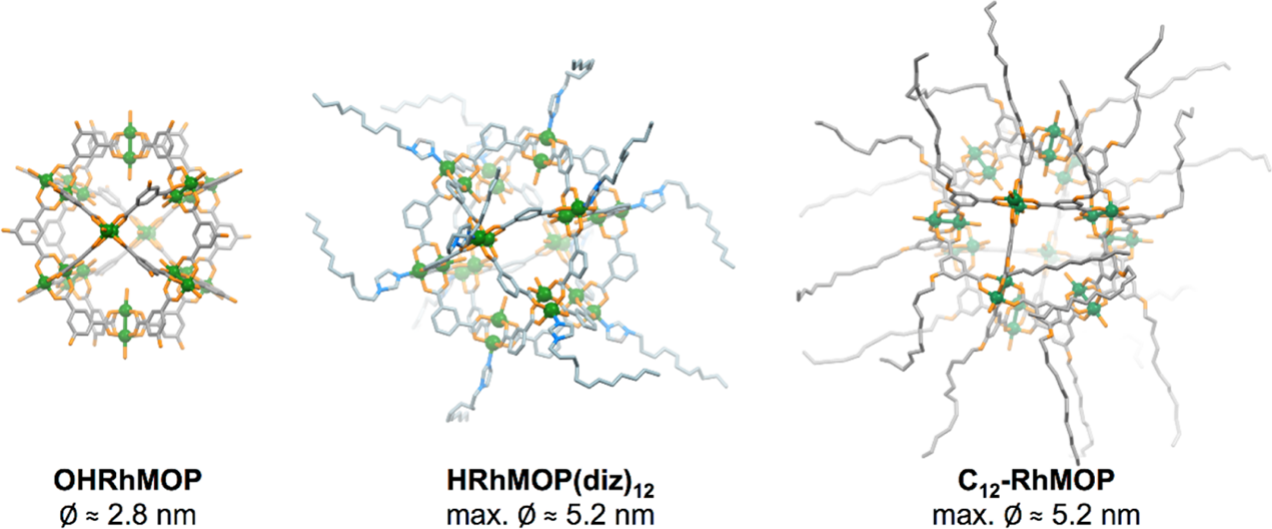
Schematic representation of the three types of Rh-MOPs included in this study: without alkyl substituents (left, OHRhMOP), with 12 alkyl chains added through coordination of an imidazole ligand at the outer axial position of each Rh pair (middle, HRhMOP(diz)_12_), and with 24 alkyl chains covalently anchored on the organic ligand (right, C_12_RhMOP). Representations are, respectively, based on the reported X-ray structures of OHRhMOP and HRhMOP(tertPy)^14^ and on the Cu analogue to C_12_RhMOP.^20^ The structure of the other Rh-MOP studied (HRhMOP(oiz)_12_) is similar to that of HRhMOP(diz)_12_, although the alkyl chains are shorter (octyl instead of dodecyl). The maximum diameter of the alkyl-functionalized MOPs was calculated considering all the alkyl chains in an extended configuration.

We initially studied the monolayer film formation of our soluble Rh-MOPs (see Figure 1) to unveil the impact of surface functionalization on their self-assembly behavior at the air–liquid interface.

**Figure 2:**
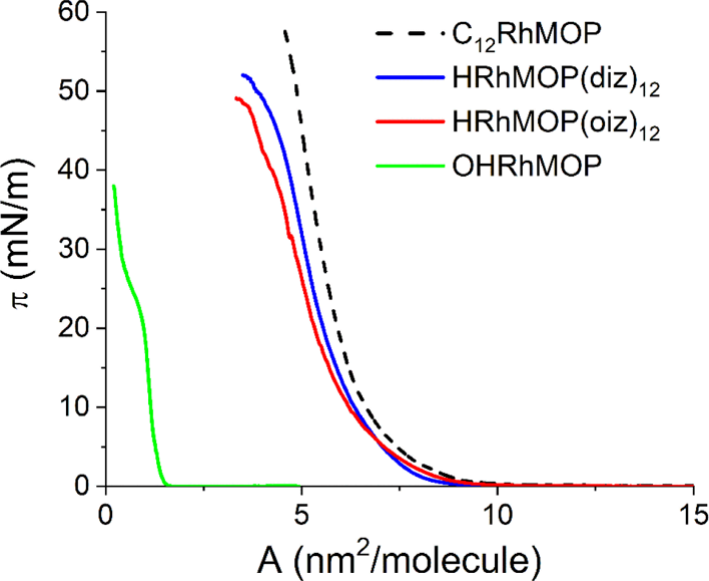
Representative surface pressure–area (π–*A*) isotherms obtained for the four Rh-MOPs studied. The C_12_RhMOP isotherm^15^ is plotted for comparison purposes (dashed line).

To this end, the appropriate volume for each compound was spread on the water subphase. The floating films obtained after solvent evaporation were compressed at a constant speed in order to obtain the characteristic surface pressure–area isotherms (π–*A*) for each compound (Figure 2).

π–*A* isotherms show a similar behavior for all Rh-MOPs functionalized with alkyl chains, including the compound C_12_RhMOP previously reported by us^15^ (plotted as the dashed line in Figure 2 for comparison). For these compounds, the surface pressure starts to rise at areas per molecule close to 9 nm^2^/molecule, and further compression leads to a marked increase of the surface pressure, revealing the formation of condensed films. However, the surface pressure reaches higher values for the Rh-MOP functionalized with 24 covalently bounded alkyl chains (C_12_RhMOP) than for the two Rh-MOPs with 12 coordinatively attached alkyl chains (HRhMOP(diz)_12_ and HRhMOP(oiz)_12_). The areas per molecule are also higher for C_12_RhMOP than for HRhMOP(diz)_12_ and HRhMOP(oiz)_12_. These results indicate that Rh-MOPs functionalized with 12 alkyl chains assemble into denser films than those functionalized with 24 alkyl chains due to the reduced steric hindrance of the former type of Rh-MOPs. On the other side, the surface pressure for OHRhMOP starts to increase at an area per molecule close to 1.6 nm^2^/molecule and reaches the maximum slope of the π–*A* curve between 1.2 and 1.0 nm^2^/molecule. Close to 20 mN/m, the slope of the isotherm decreases and a pseudo-plateau can be observed. Finally, at areas below 0.45 nm^2^/molecule, the surface pressure increases again markedly. The areas obtained for this Rh-MOP, even at low surface pressures, are lower than the expected size of a single molecule, revealing that, possibly, the formed film is not a homogeneous monolayer.

**Figure 3:**
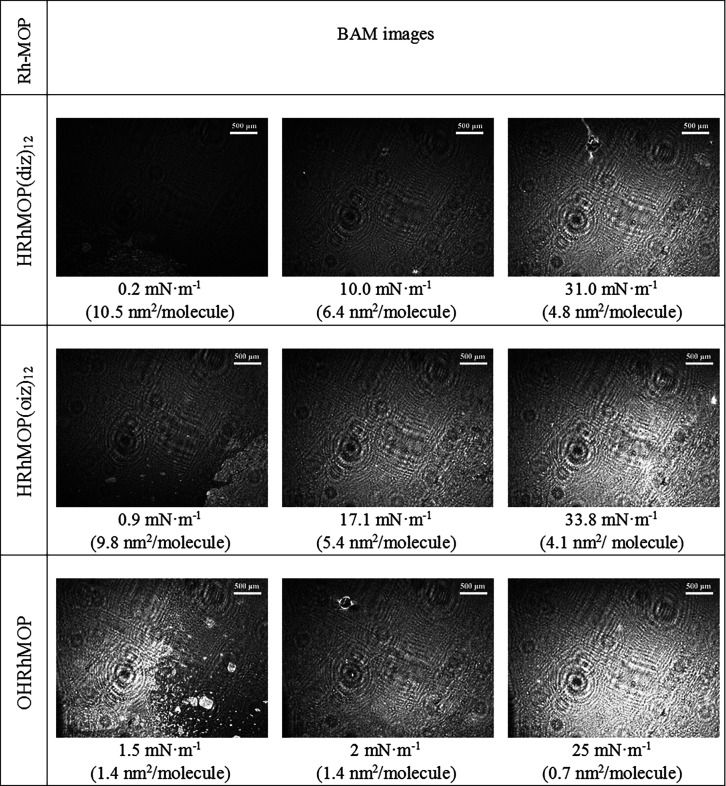
BAM images obtained during Rh-MOP film compression at indicated surface pressures and the corresponding areas per molecule.

The BAM images obtained simultaneously to π–*A* isotherms (Figure 3) show thin film domains and uncovered water regions at low surface pressures for all tested Rh-MOPs. These domains merge and form homogeneous monolayers that completely cover the water surface at surface pressures above 10 mN/m for HRhMOP(diz)_12_ and 15 mN/m for HRhMOP(oiz)_12_, while for C_12_RhMOP, it has been reported that it is necessary to increase the surface pressure up to 25 mN/m to fully cover the water surface.^15^ However, OHRhMOP film domains are brighter, which indicates that these domains are thicker than those observed for the Rh-MOPs functionalized with aliphatic chains, even at a very low surface pressure. This indicates that the floating films obtained at low pressures are not true monolayers but rather contain multilayer domains. At a low surface pressure of *ca.* 2 mN/m, these surface aggregates assemble into a film that covers the entire water phase. The BAM images obtained at this stage reveal the formation of thick films as evidenced by the brightness of the images.

**Figure 4:**
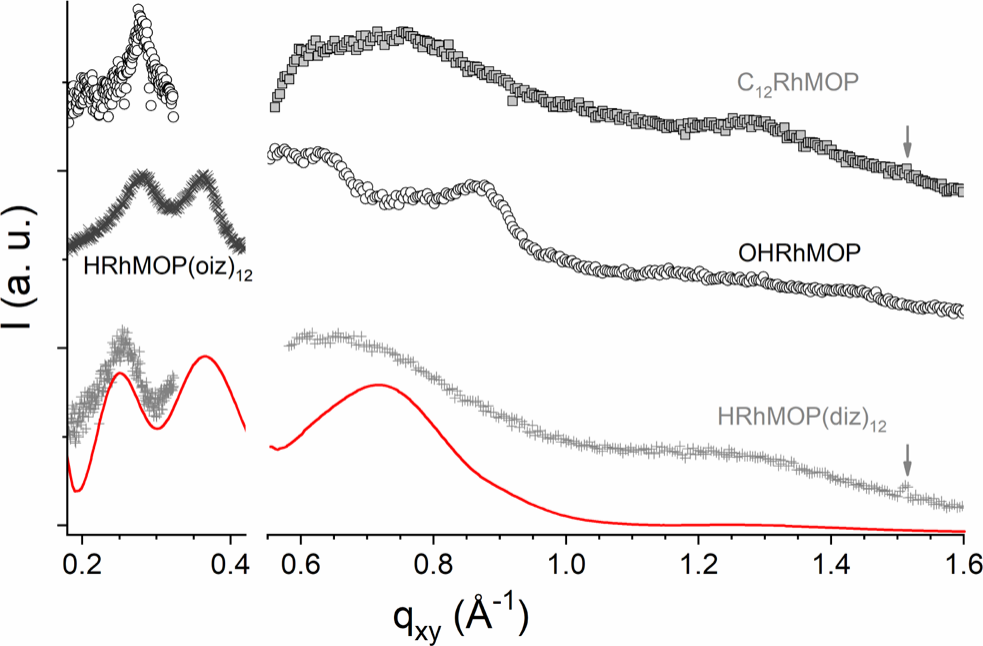
*In situ* GIXD (*q* > 0.55 Å^–1^) and GISAXS (*q* < 0.45 Å^–1^) data for the studied Rh-MOPs at the gas–water interface at 5 mN/m. Code: C_12_RhMOP (■), OHRhMOP (○), HRhMOP(diz) _12_ (+), and HRhMOP(oiz) _12_ (×). The data have been shifted vertically for clarity. The red line is the diffusion form factor of core–double shell spheres with an empty (SLD = 0) core of 5 Å radius, a first dense shell of 10.5 Å thickness (SLD = 2 × 10^–6^ Å^–2^), and an outer less dense shell of 18 Å thickness, considering a pinhole instrumental smearing δ Q/Q of 10%. This model was calculated using SasView.^22^ Note that it has been scaled differently for the two data ranges. Vertical arrows highlight the peak at 1.51 Å^–1^ corresponding to alkyl chain ordering.

In order to further investigate the behavior of Rh-MOPs at the air–water interface and determine if the assembled continuous films show some regular crystalline arrangement, GIXD and GISAXS studies were performed. The two Rh-MOPs with dodecyl chains, C_12_RhMOP and HRhMOP(diz)_12_, and that with 24 hydroxo groups, OHRhMOP, were first studied in GIXD configuration (*q* range 0.55–1.8 Å^–1^). Very similar results are obtained for the two Rh-MOPs with dodecyl chains, at both tested pressures (5 and 10 mN/m) and after collapse (Figures 4 and S2). For C_12_RhMOP and HRhMOP(diz)_12_, two very broad humps are observed at *ca.* 0.76/0.71 and 1.29/1.21 Å^–1^, respectively. These are likely resulting from the diffusion form factors of the Rh-MOPs, which in first approximation can be considered as core–shell spheres. Indeed, a reasonably good simulation can be obtained with either a core–shell or a core–double shell model and the characteristics of the Rh-MOPs. In addition, a sharp peak is observed systematically for both C_12_RhMOP and HRhMOP(diz)_12_ at *q* = 1.51 Å^–1^, characteristic of ordered alkyl chains.^21^ Interestingly, the peak arises solely from in-plane scattering (Figure S3), thus indicating that the chain organization *a priori* takes place parallel to the subphase. Indeed, the Bragg rod has only a very limited *q*_*z*_ extension (Figure S3), in disagreement with the form factor of dodecyl chains^21^ or other classical amphipathic molecules that extend perpendicular to the water subphase. This in turn suggests that Rh-MOP molecules are forming a film in which they are softly connected through interdigitation of some of their dodecyl chains. An additional weaker peak at *ca.* 1.68 Å^–1^ is also detected in the collapsed film for C_12_RhMOP, which could indicate a herringbone arrangement of the dodecyl chains. The fact that this peak is not observed in the case of HRhMOP(diz)_12_ may be ascribed to its lower density of dodecyl chains (12 vs 24).

The GIXD data obtained for OHRhMOP are similar for the three pressures studied (1, 5, and 10 mN/m) but differ significantly from those of the Rh-MOPs with dodecyl chains (Figures 4 and S2). Four broad peaks are clearly distinguished at *ca.* 0.57, 0.63, 0.77, and 0.87 Å^–1^, in addition to several weaker and poorly resolved features in the 1.1–1.5 Å^–1^ range. While the diffusion form factor for a core–shell sphere of the characteristics of OHRhMOP does coincide with the two stronger peaks at 0.63 and 0.87 Å^–1^ (Figure S4), the rest of features, and in particular the peaks at 0.57 and 0.77 Å^–1^, cannot be reasonably predicted by this model. This could mean that, in the case of OHRhMOP, the observed features are also in part resulting from a regular organization of the Rh-MOPs. The corresponding higher order peaks are unfortunately not scanned by GIXD due to the high background at low *q*. Nevertheless, GISAXS data for OHRhMOP do present a relatively sharp peak at 0.28 Å^–1^, which is in reasonably good agreement with the expected Bragg peak arising from a close-packed arrangement of OHRhMOP, which has a size of *ca.* 28 Å. The peak at 0.57 Å^–1^ in GIXD can thus be ascribed to the second order, altogether supporting the presence of domains of regularly organized OHRhMOP.

HRhMOP(diz)_12_ and the Rh-MOP with shorter octyl chains HRhMOP(oiz)_12_ were also studied in GISAXS configuration. For both Rh-MOPs with alkyl chains, no low *q* peak is detected that could correspond to the inter-MOP separation *D* in a crystalline arrangement, typically expected in the range 0.14–0.18 Å^–1^ considering Rh-MOP sizes in the range 40–52 Å in a close to hexagonal packing and thus *D* = 2π/*q*_*xy*_(√3). This supports the assumption that homogeneous films formed by Rh-MOPs with alkyl substituents do not present crystalline organization. Furthermore, two broad peaks are detected in the range of 0.27–0.42 Å^–1^ (see Figure 4). These peaks are reasonably reproduced, together with those at higher *q*, considering the diffusion form factor of core–double-shell spheres with an empty core (calculation made using SasView^22^); that is, with zero scattering length density (SLD), of 5 Å radius, a first dense shell of 10.5 Å, thickness corresponding to the Rh-ligand structure (SLD = 2 × 10^–6^ Å^–2^), and an outer less dense shell of 18 Å thickness corresponding to the alkyl arms (SLD = 0.6 × 10^–6^ Å^–2^). This is in line with the characteristic sizes of both Rh-MOPs, which have a core of *ca.* 28 Å diameter and a total size of up to 52 Å diameter considering extended dodecyl chains (and thus, a shell thickness of up to 24 Å). It is worth noting that the broad humps at *ca.* 0.4 and 0.7 Å^–1^ are in good agreement with SAXS data obtained on Cu(II) analogues of C_12_RhMOP with varying alkyl chain lengths,^16^ thus supporting our model. On the contrary, our experimental observation of in-plane ordering of some of the alkyl chains in films of C_12_RhMOP and HRhMOP(diz)_12_*a priori* contradicts the alkyl chain/Rh-MOP cores/alkyl chains three-layer model used to simulate the NR data, albeit limited to *q* < 0.1 Å^–1^, of the LB film of C_18_-CuMOP.^16^

Altogether, the GIXD/GISAXS data supports the fact that Rh-MOP molecules with alkyl chains form a sort of glassy film at the air–water interface, with the Rh-MOPs maintaining motional degrees of liberty, thus having variable orientations and inter-MOP separations. The film is formed at rather low pressure, probably as soon as inter-MOP interactions are favored, through interdigitation of the alkyl chains. Although the chains show some in-plane order, this does not result in a regular organization of the Rh-MOPs in the film. In the case of OHRhMOP, the experimental scattering data is likely the sum of diffraction arising from a crystalline arrangement in multi-layer Rh-MOP aggregates, as pointed out by BAM observations and diffusion from Rh-MOPs in other, unorganized, areas of the film.

### 3.3 Rh-MOP Film Deposited onto Solid Substrates

Having characterized the Rh-MOP behavior at the air–liquid interface, Rh-MOP films have been transferred onto solid substrates. The first step was the optimization of the experimental conditions for the sequential deposition of homogeneous monolayers in order to build Rh-MOP multilayers with the desired thickness onto different substrates.

Previous studies carried out with C_12_RhMOP showed that LS Rh-MOP films can be used, for instance, as selective materials in composite membranes for CO_2_ separation.^15^ LS deposition is especially suitable for this application since it is faster than LB deposition (which is highly time consuming for the deposition of a large number of layers), and the selective film is deposited only on one side of the porous support, which is required to obtain asymmetric composite membranes.

In this work, the LS films of HRhMOP(diz)_12_ and HRhMOP(oiz)_12_ were first transferred onto quartz substrates at 20 mN/m, whereas OHRhMOP LS films were deposited at 2 mN/m. All the films were characterized by AFM and UV–vis spectroscopy.

**Figure 5:**
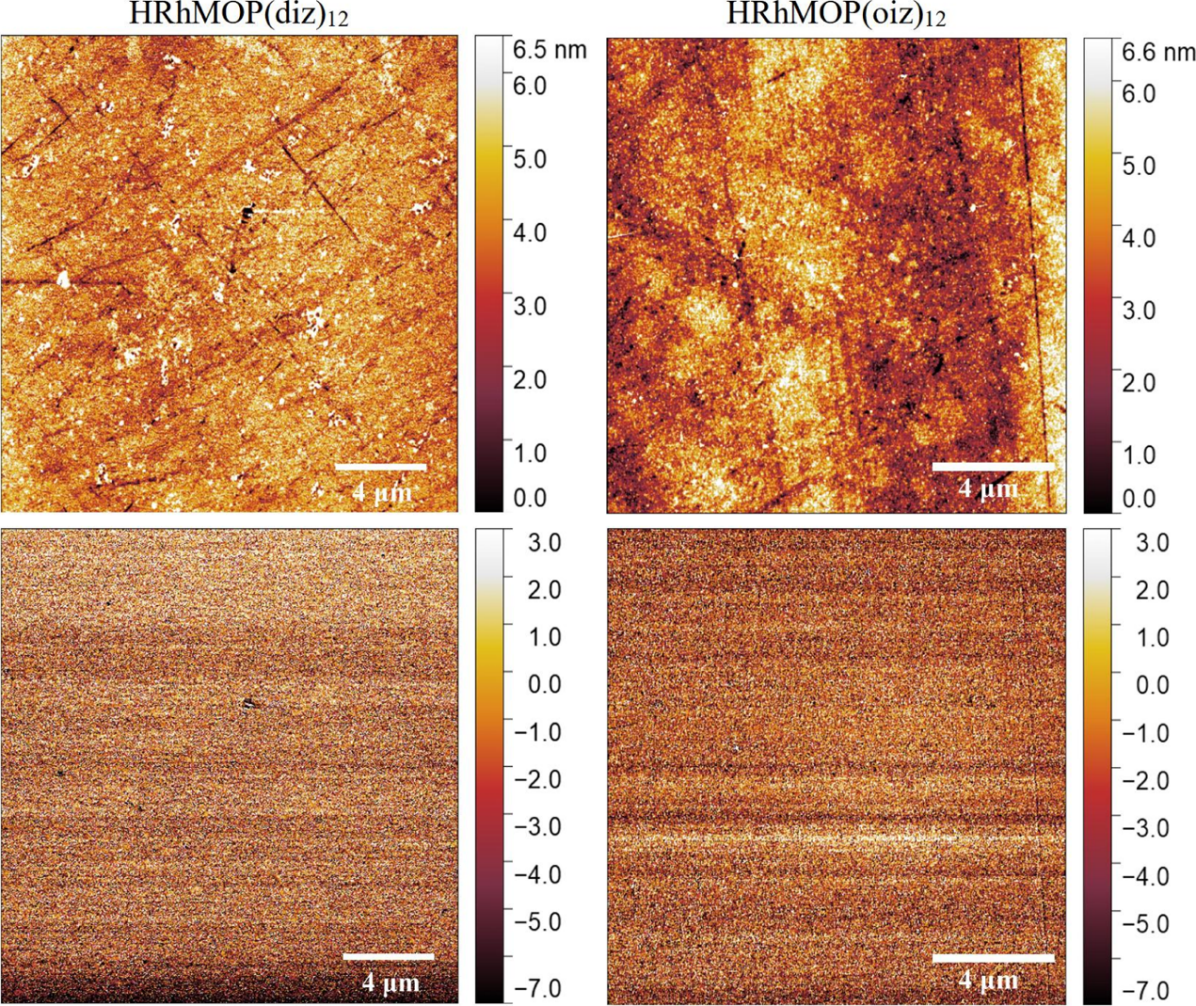
Representative AFM topography (top) and phase images (bottom) from HRhMOP(diz)_12_ and HRhMOP(oiz)_12_ LS films transferred onto quartz substrates at 20 mN/m. The observed scratches correspond to the defects on the surface of the quartz plates used (see Figure S7) since phase images confirm the homogeneity of the samples.

The comparison of UV–vis spectra for LS monolayer films of HRhMOP(diz)_12_ and HRhMOP(oiz)_12_ with those of dichloromethane solutions (Figure S5) shows a very good agreement between both types of films and solution spectra at wavelengths above the solvent cut-off, which confirms that film formation does not affect the integrity of the materials. Additionally, AFM characterization of LS monolayers of HRhMOP(diz)_12_ and HRhMOP(oiz)_12_ (Figure 5) shows flat and homogeneous films that completely cover quartz substrates at 20 mN/m. The average thickness of these films was estimated by measuring different film defects and borders with the AFM tip (Figure S6). HRhMOP(diz)_12_ monolayers are *ca.* 2.5 nm thick, similarly to C_12_RhMOP films, whereas HRhMOP(oiz)_12_ monolayers are slightly thinner (*ca.* 2.3 nm), which is related to the shorter alkyl chain length for this compound. The root-mean-square (rms) roughness of MOP monolayers was also evaluated using topography images. HRhMOP(diz)_12_ and HRhMOP(oiz)_12_ films present a similar rms roughness data (0.95 and 0.86 nm, respectively). Remarkably, these values are even lower than the rms roughness of the uncovered quartz substrates used for film deposition (Figure S7), which is close to 1.3 nm, due to the presence of defects on the surface of the quartz plates.

The OHRhMOP LS films transferred onto quartz substrates show similar spectra to those in solution (Figure S5). However, the absorbance of the film is significantly higher than the absorbance of the monolayer films of HRhMOP(diz)_12_ and HRhMOP(oiz)_12_. Moreover, AFM characterization (Figure S8) shows films with an average thickness of *ca.* 3 nm and an rms roughness of *ca.* 2.3 nm due to the presence of several defects of lateral size between 250 and 400 nm and heights between 30 and 50 nm, which confirms that this Rh-MOP without alkyl chains does not form homogeneous monolayers at the air–water interface.

**Figure 6:**
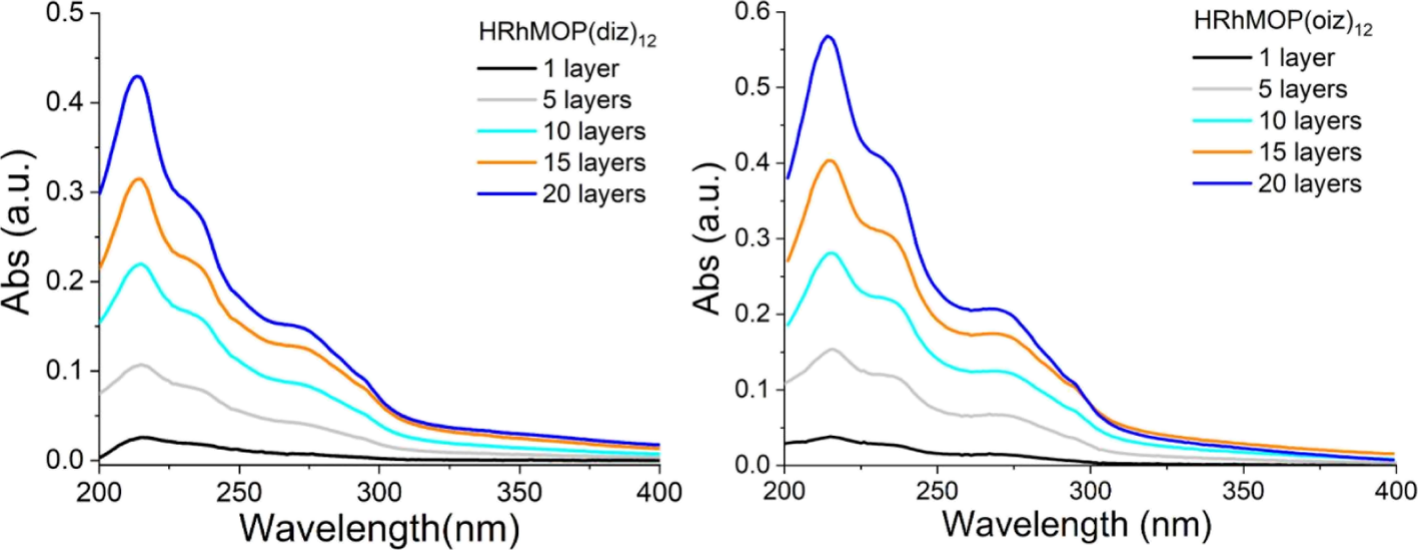
UV–vis spectra from HRhMOP(diz)_12_ and HRhMOP(oiz)_12_ sequential deposition of LS films transferred onto quartz at 20 mN/m.

The sequential deposition of multilayer films of HRhMOP(diz)_12_ and HRhMOP(oiz)_12_ at 20 mN/m onto quartz and quartz crystal microbalance disks was then investigated. Figure 6 shows the continuous increment of the film absorbance with the number of Rh-MOP monolayers deposited, without altering the position of the absorption bands. Moreover, the absorbance at the wavelength of maximum absorption (*ca.* 215 nm) was plotted against the number of LS monolayers deposited, showing a linear increase of the absorbance with the number of deposited layers, which demonstrates an almost constant Rh-MOP deposition in each transfer (Figure S9).

Rh-MOP films deposited onto QCM disks were used to determine the correlation between the deposited mass and the number of monolayers transferred (see Figure S10). From the slopes, almost constant mass depositions were obtained for each Rh-MOP monolayer transferred of HRhMOP(oiz)_12_ (0.32 μg/cm^2^) and HRhMOP(diz)_12_ (0.35 μg/cm^2^). These values give a similar mean molecular area for both compounds in LS films (*ca.* 4.4 nm^2^/molecule), which is smaller than the areas obtained for the same Rh-MOPs at the air–water interface (around 5.4 nm^2^/molecule). The area obtained from C_12_RhMOP multilayer films was significantly higher (5.8 nm^2^/molecule). Moreover, for this compound, the area was very close to the value at the air–water interface. This points to a densification of multilayer films deposited onto solid substrates for the Rh-MOPs with 12 alkyl chains, whereas the films of the Rh-MOP with 24 alkyl chains retain almost exactly the same superficial density obtained at the air–liquid interface. These results, which are in line with the observation derived from the π–*A* isotherms, further support the relationship between film density and the extent of aliphatic functionalization on the surface of the Rh-MOP.

### 3.4 Air–Water Interfacial Formation of Alkyl-Functionalized Rh-MOP Films

Preceding sections have shown that *ex situ* aliphatic chain functionalization on the surface of Rh-MOPs enables their processing into ultrathin films through the LB technique. Such aliphatic chains can be incorporated on the surface of the Rh-MOP before the formation of the film through ligand design (C_12_RhMOP) or through coordinative post-synthetic modifications (HRhMOP(oiz)_12_ and HRhMOP(diz)_12_).

**Figure 7:**
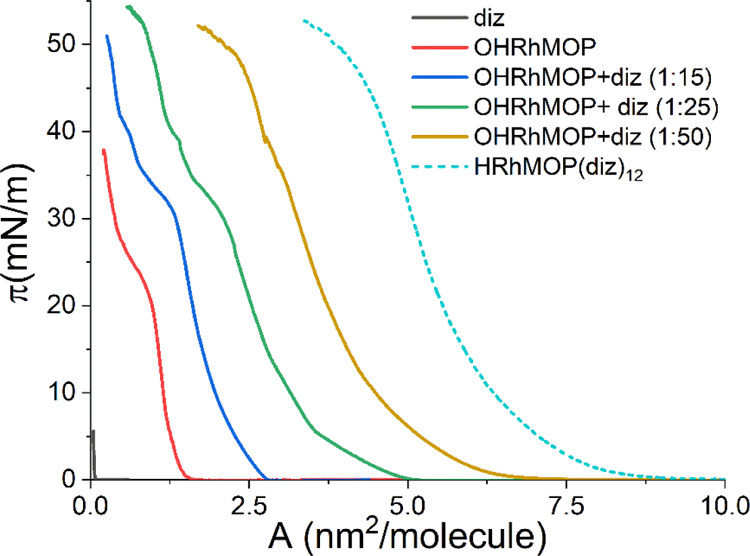
Surface pressure–area (π–*A*) isotherms obtained for the imidazole ligand (diz) and different OHRhMOP/diz proportions (for these systems, the area per molecule is calculated considering only the amount of Rh-MOP molecules). A HRhMOP(diz)_12_ isotherm has also been plotted for comparison (dashed line).

On the other hand, OHRhMOP showed to aggregate upon film formation. We envisaged that the degree of aggregation of OHRhMOP could be controlled by functionalizing OHRhMOP films *in situ* during film formation. With this aim, different amounts of separate solutions of the diz ligand and OHRhMOP were spread successively onto the air–water interface. Figure 7 shows some representative π–*A* isotherms obtained in this study. The diz ligand almost does not show any activity at the air–water interface, and the surface pressure only rises slightly at very small surface areas, revealing that diz alone cannot form stable Langmuir films.

Given that the stoichiometric relationship OHRhMOP/diz is 1:12 if all the axial sites are occupied by imidazole ligands, different OHRhMOP/diz proportions were tested. As shown in Figure 7, a significant increase of the area per Rh-MOP molecule, compared to pure OHRhMOP, is obtained when 15 diz ligands per OHRhMOP are spread on the water surface. However, the areas per Rh-MOP are still far from the values obtained for alkyl-functionalized Rh-MOPs. When the amount of diz is increased up to 25 or 50 diz ligands per OHRhMOP, the area per Rh-MOP is also increased almost proportionally to the increment of diz amount. Moreover, the shape of these isotherms is quite similar to the isotherm of HRhMOP(diz)_12_, which seems to indicate that the chemical reaction at the air–water interface leads to the formation of an alkyl-functionalized Rh-MOP in a similar manner as observed in solution. BAM images were also acquired during film compression (Figure S11), revealing the formation of dense films at surface pressures above 15 mN/m, although more defects were observed when 50 diz ligands per OHRhMOP are spread. Consequently, the proportion 1:25 was chosen for further studies.

**Figure 8:**
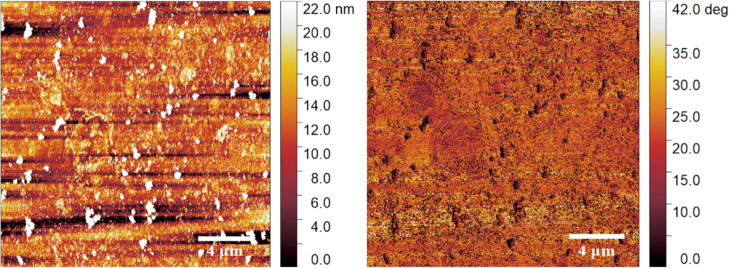
Representative AFM topography and phase images from an OHRhMOP + diz LS film transferred at 20 mN/m onto a quartz substrate. The film was prepared using a 1:25 OHRhMOP/diz proportion at the air–liquid interface.

The films obtained after spreading 25 diz ligands per OHRhMOP molecule were transferred by LS deposition onto different substrates at 20 mN/m. Figure 8 shows a representative AFM image of one LS film transferred onto a quartz substrate. A continuous film with a thickness of *ca.* 2.8 nm is obtained, although some aggregates of height up to 200 nm and lateral size between 400 and 600 nm can also be observed. A plausible explanation is that these aggregates could be formed by unreacted OHRhMOP since this Rh-MOP has a tendency to self-aggregate at the air–water interface as shown in the previous characterization of OHRhMOP films.

**Figure 9:**
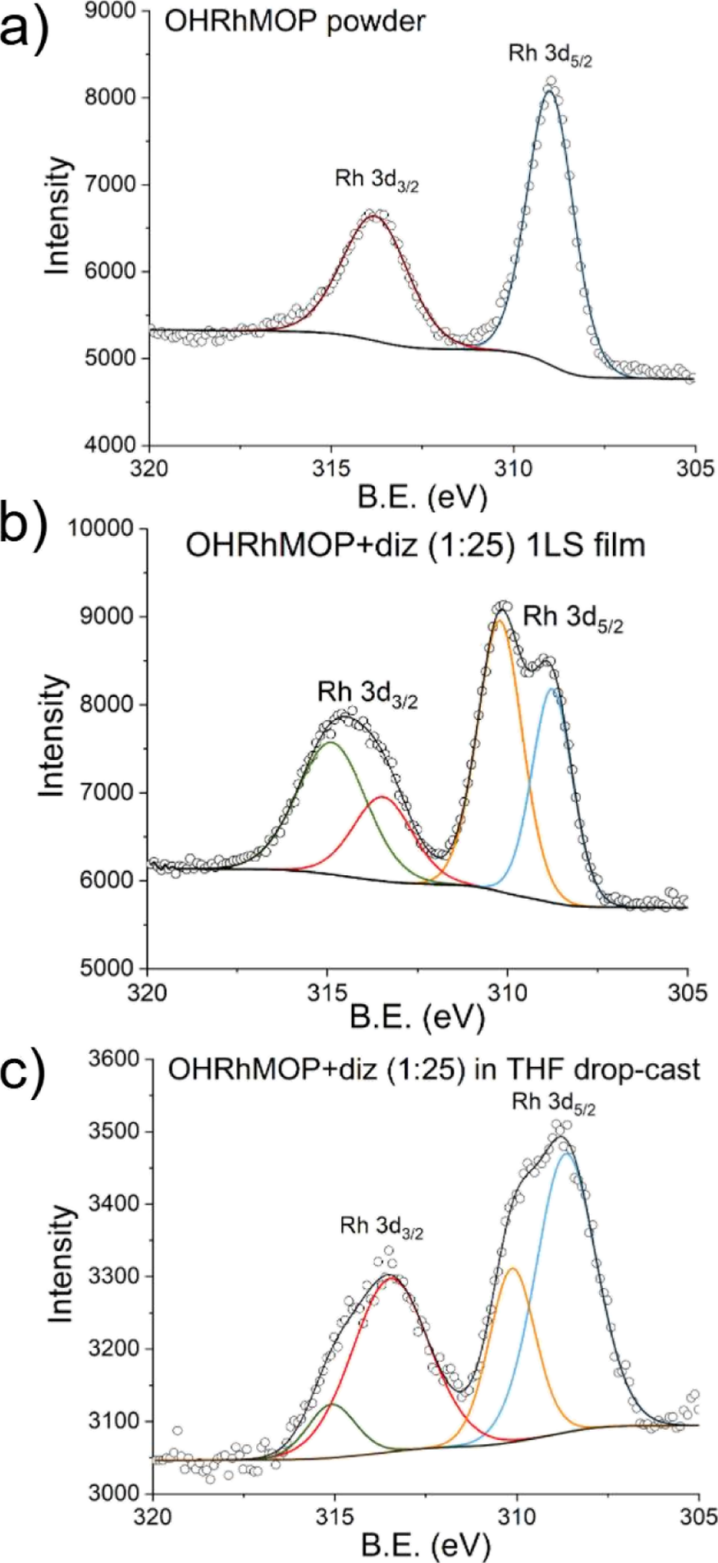
XPS spectra of (a) OHRhMOP (powder), (b) 1 LS film deposited at 20 mN/m after OHRhMOP + diz (1:25) reaction at the air–liquid interface, and (c) drop-cast film obtained after OHRhMOP + diz (1:25) reaction in THF. The black lines represent the simulation of these spectra using CasaXPS and a Shirley-type background. The components depicted as colored lines are detailed in Table S1.

Additional characterization of LS films transferred onto Si(100) substrates was made by XPS. Figure 9 presents the comparison of the XPS spectra of solid OHRhMOP and alkyl-functionalized Rh-MOP films obtained by the OHRhMOP reaction with excess of diz (1:25 proportion) in THF or at the air–water interface. Previous literature studies^23^ indicate that the Rh 3d high-resolution spectrum is composed of a doublet peak (3d_5/2_ and 3d_3/2_) with a spin–orbital coupling energy difference of 4.74 eV. The OHRhMOP spectrum shows these peaks at *ca.* 309.0 and 313.8 eV, in good agreement with the literature. However, the samples obtained after MOP reaction with excess diz present broader peaks, and the deconvolution of these peaks allows obtaining some interesting conclusions. The 3d_5/2_ peak deconvolution shows two contributions at *ca.* 308.7 and 310.1 eV, while the deconvolution of the 3d_3/2_ peak results in two bands at *ca.* 313.5 and 315.0 eV. The bands at 308.7 and 313.5 eV are reasonably close to the peaks obtained for OHRhMOP and can be ascribed to unmodified Rh paddlewheels, while the new peaks at *ca.* 310.1 and 315 eV present a significant shift of *ca.* 1.1 eV to a higher binding energy, which is likely a consequence of diz ligand coordination to Rh. Indeed, diz is a stronger donor than the O-donors, either H_2_O or MeOH, likely occupying the axial position of Rh paddlewheels in OHRhMOP.

Interestingly, the relative amount of Rh atoms with modified 3d bands is higher in the film obtained at the air–liquid interface, although the solution spectra obtained in THF confirm that all the Rh paddlewheels are coordinated to diz in solution. A plausible explanation to this fact can be the evaporation of THF after drop-cast deposition, which results in a partial uncoordination of diz ligands (or exchange with water molecules from the air) in the unstructured film, while LS films are densely arranged, preventing diz uncoordination. Overall, XPS measurements confirm the *in situ* coordination of diz to OHRhMOP at the water–air interface.

Then, multilayer LS films of the interfacially functionalized Rh-MOP were fabricated by sequential deposition of Langmuir films onto quartz and QCM substrates. After each deposition, the samples were washed with hexane with the aim of eliminating, at least partially, the observed aggregates without damaging the Rh-MOP film. Figure S12 shows the continuous increment of the film absorbance and the surface mass density with the number of layers deposited, respectively, onto quartz and QCM substrates. These results are very similar to the behavior observed for alkyl-functionalized Rh-MOPs. In particular, the increment of the absorbance per deposited layer is almost the same than for HRhMOP(oiz)_12_, although the wavelength of the maximum absorbance is slightly shifted to 221 nm. Meanwhile, the deposited mass increases up to 0.44 μg/cm^2^ probably due to the unreacted OHRhMOP aggregates and diz chains that are present on the films and the denser structure of the films derived from the π–*A* isotherm.

### 3.5 Post-Synthetic Modification of Alkyl-Functionalized Rh-MOP Films

We have shown that it is possible to functionalize Rh-MOP films at the air–water interface coordinating a N-donor ligand (diz) to Rh dimers’ axial sites through an interfacial reaction. However, the reversible nature of the interaction between diz and the Rh-MOP suggests that it should also be possible to post-synthetically detach diz ligands from previously assembled HRhMOP(diz)_12_ films. With this aim, LS films of different thicknesses (one and three layers) of HRhMOP(diz)_12_, deposited onto quartz and Si(100) substrates, were exposed to HCl vapors at room temperature and subsequently washed with *n*-hexane. The acid treatment was selected because it has been previously employed with success to detach N-donor ligands from Rh-MOPs in solution.^24^ The acid-treated films were characterized through UV adsorption spectra, water contact angle (WCA), and AFM to analyze the chemical and morphological changes induced by acid treatment.

Table 1 presents the WCA values of LS films deposited onto Si(100) and quartz substrates before and after the treatment with HCl vapor. The deposition of HRhMOP(diz)_12_ ultrathin LS films significantly increases the hydrophobicity of the substrates, reaching values around 91° on Si and 88° on quartz substrates, which suggest an efficient packing of the alkyl chains from diz ligands on the upper side of Rh-MOP films. Only 2 min after being exposed to HCl vapors, the WCA of these samples diminish between 10 and 18°. Longer treatment (30 min) does not change significantly the WCA values, except in the case of the monolayer film deposited on quartz that reduces to *ca.* 57°. These results reveal a decrease of film hydrophobicity, which can be consistent with a partial removal of diz ligands from the film.

**Table 1:** WCA Values (Average ± Standard Deviation) for Ultrathin Films of HRhMOP(diz)_12_ Transferred onto Si(100) or Quartz Substrates^a^

	WCA			
			film after HCl treatment	
sample	uncovered substrate	pristine film on the substrate	*t* = 2 min	*t* = 30 min
1 layer Si(100)	44.9 ± 0.4	91.6 ± 0.2	77.8 ± 0.6	76.0 ± 0.2
3 layers Si(100)		91.2 ± 0.2	73.4 ± 0.3	73.1 ± 0.4
1 layer quartz	15.4 ± 0.6	80.7 ± 0.2	72.9 ± 4.3	56.8 ± 0.5
3 layers quartz		88.1 ± 0.3	78.1 ± 0.6	74.4 ± 1.4

a For comparison purposes, uncoated substrates were also analyzed.

The comparison of UV spectra of the samples with one monolayer deposited onto a quartz substrate before and after HCl treatment shows that the band of maximum absorption at *ca.* 215 nm is not affected by the acid treatment, which seems to indicate that the MOP polyhedral core surface density is similar after the acid treatment (Figure S13). Note that the detachment of diz from the Rh-MOP films could not be monitored by UV–vis. The π* → σ* transition of the Rh(II) paddlewheel (band I), which is sensitive to the coordination environment of the Rh(II) axial site, is a forbidden transition with low molar attenuation coefficient.^25^ The minute amount of deposited Rh-MOP on the substrate makes not possible to analyze the changes of band I on these samples.

Finally, the AFM images of a monolayer film deposited onto Si(100) before and after acid treatment for 30 min were also obtained (Figure S14). Phase images show that the surface coverage is similar after acid treatment, although more pinholes are present in the film, as can be observed in the topography images. However, these defects scarcely modify the rms roughness of the samples, which increases from 0.29 to 0.41 nm (uncovered Si(100) substrates present a rms roughness of 0.26 nm, see Figure S7).

Altogether, these results point to a cleavage of the diz ligands exposed to the air by acid treatment, while the interdigitated alkyl chains are not affected, thus keeping a similar film coverage after the acid treatment. Therefore, we have shown that thin films made by coordinatively functionalized Rh-MOPs can be post-synthetically modified to tune their hydrophobicity, which in turn might impact on the film applications.

### 3.6 Rh-MOP Films in CO_2_/N_2_ Separation

**Figure 10:**
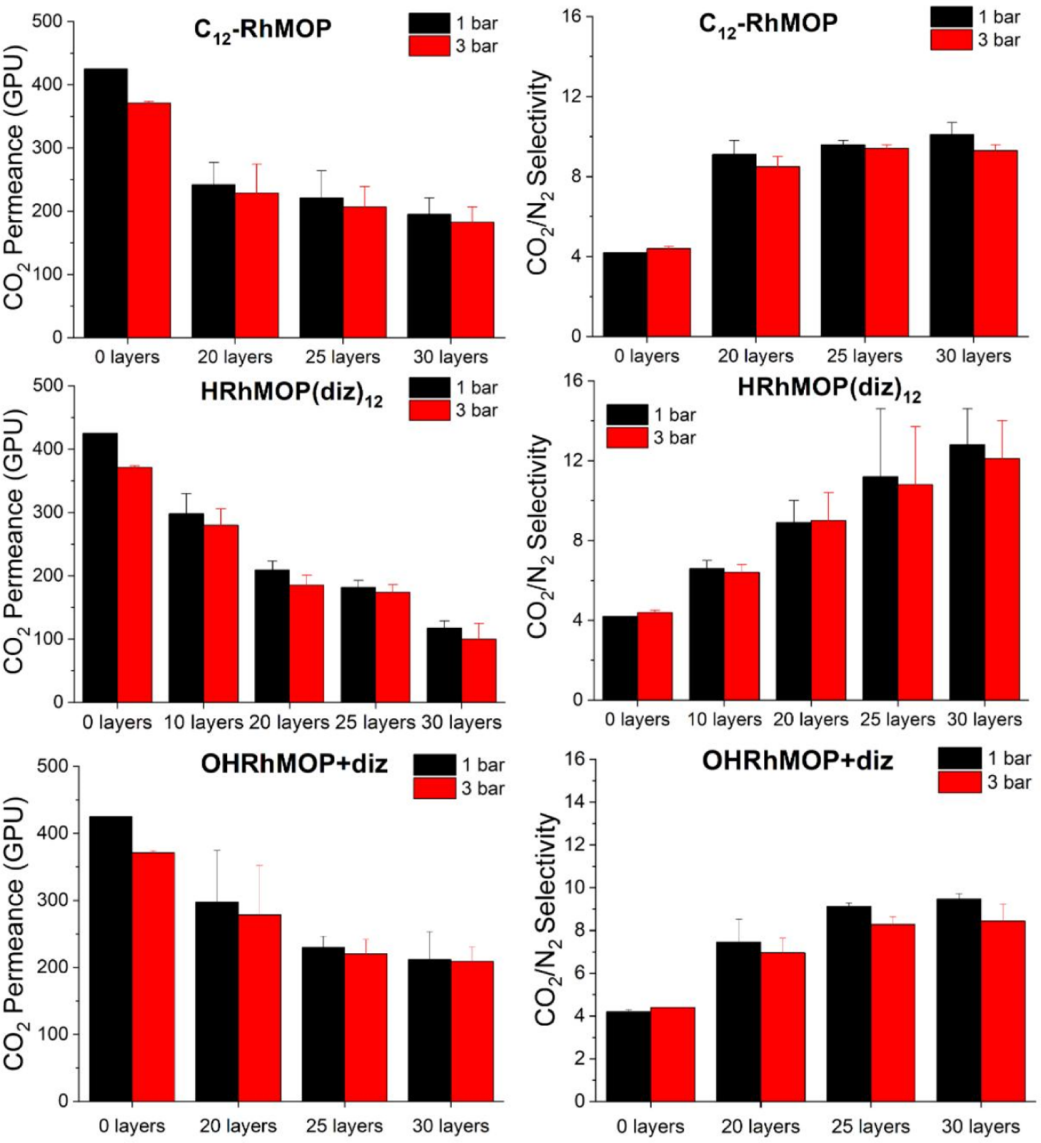
CO_2_ permeance and CO_2_/N_2_ selectivity measurements of HRhMOP(diz)_12_ and OHRhMOP + diz samples transferred onto PTMSP. Measurements were made at 35 °C and at two different pressures (1 and 3 bar). Error bars were obtained from measurements on at least two different samples. C_12_RhMOP data previously reported by us^15^ have also been included for comparison.

HRhMOP(diz)_12_ LS multilayer films were deposited onto the permeable polymer PTMSP in order to fabricate selective Rh-MOP layers with the desired film thickness. Here, multilayer films formed by 10–30 Rh-MOP monolayers were tested for CO_2_/N_2_ separation in post-combustion conditions (temperature 35 °C, feed pressures 1 and 3 bar, and CO_2_/N_2_ mixture composition 10/90 in volume). CO_2_ permeance and CO_2_/N_2_ selectivity average values are shown in Figure 10. The CO_2_/N_2_ selectivity of the PTMSP membrane at 1 bar is 4.1 and constantly increases with the deposition of HRhMOP(diz)_12_ films up to values close to 13 when 30 Rh-MOP monolayers are deposited, while the CO_2_ permeance diminishes from 415 to *ca.* 120 GPU (at 1 bar). Comparing the performance of HRhMOP(diz)_12_ membranes to the values obtained for C_12_RhMOP in the same conditions, the higher CO_2_/N_2_ selectivity values obtained for HRhMOP(diz)_12_ samples can be highlighted (maximum values for 30 monolayer films of C_12_RhMOP are close to 10), which logically leads to lower CO_2_ permeance values (*ca.* 195 GPU for C_12_RhMOP). These results are in good agreement with the denser packing of the HRhMOP(diz)_12_ films discussed in previous sections and pave the way for the modulation of selectivity and permeance of MOP films in gas separations by controlling the number of alkyl chains attached to a given MOP polyhedral core.

Additionally, multilayer films of the interfacially modified OHRhMOP with diz ligands were also deposited onto PTMSP substrates in order to investigate its performance in CO_2_/N_2_ separation. Figure 10 shows the CO_2_ permeance and CO_2_/N_2_ selectivity average values. The CO_2_/N_2_ selectivity increases with the number of layers deposited at the same time that the CO_2_ permeance diminishes, similarly to the results obtained for alkyl-functionalized MOPs. However, in this system, the changes between the samples containing 25 or 30 layers are more limited, and the deviations between different samples incorporating the same number of layers are smaller with 25 than with 30 layers. Interestingly, the CO_2_ permeance values at 1 and 3 bar are almost identical for the membranes obtained with the interfacially functionalized Rh-MOP and bigger than the permeances obtained for alkyl-functionalized Rh-MOPs, maintaining CO_2_/N_2_ selectivities similar to that of C_12_RhMOP. This confirms that the interfacial reaction between diz ligands and MOPs that do not possess alkyl chains is an efficient method to obtain ultrathin MOP films that can be incorporated onto different substrates and opens an interesting methodology for the development of MOP-based films with different structures and functionalities.

Although the performances of the MOP films fabricated herein are below the commercial target for post-combustion CO_2_ capture,^26^ it should be highlighted that they have been evaluated for the separation of CO_2_ from N_2_ under experimental conditions relevant to post-combustion CO_2_ capture (10% CO_2_ and 90% N_2_ and two different feed pressures, 1 and 3 bar). Moreover, the performances of MOP films are comparable to those previously reported for LS films fabricated with different polymers of intrinsic microporosity (PIMs),^19^ as shown in Table S2. In particular, the performance of HRhMOP(diz)_12_ films is very similar to that of PIM films, both in terms of CO_2_ permeance and CO_2_/N_2_ selectivity. Also, C_12_RhMOP and interfacially functionalized MOP films present larger CO_2_ permeances than PIM films (up to 1.8 times larger), which leads to lower CO_2_/N_2_ selectivity values. Consequently, we have shown that the approach introduced in this study allows fabricating dense MOP films with tailorable performance in CO_2_/N_2_ separation. Moreover, the LB technique is the only method reported up to the date that allows obtaining pure MOP selective layers that can be used for gas separation.

## 4 Conclusions

The formation and deposition of ultrathin films of Rh-based MOPs through the LB technique have been successfully expanded to Rh-MOPs bearing alkyl substituents introduced through coordination at the Rh paddlewheel external sites, instead of those with substituents on the organic ligand of the Rh-MOP previously reported. The obtained films are dense and homogeneous monolayers, resulting from partial interdigitation of alkyl chains. *In situ* modification of Rh-MOP films can be done by either partially grafting alkyl substituents through coordination at the air–water interface or by partially removing these through exposure of pre-formed films to acid vapors. Multilayered films of Rh-MOPs with alkyl substituents all act as selective layers for efficient CO_2_ separation, including those obtained by interfacial modification, thus opening a useful path to variations of MOP film structure and functionality. Overall, the results presented here enable to expand the composition of MOP-based films by incorporating previously inaccessible MOPs (*i.e.*, insoluble MOPs) and by post-synthetic modification of the MOP-based films.

## Supporting Information Available

UV–vis characterization of OHRhMOP alkyl functionalization with diz in solution; GIXD characterization during MOP film formation; schematic representation of MOP LS film deposition; UV–vis and AFM characterization of MOP films deposited onto quartz substrates; Rh-MOP mass deposited onto QCM disks; BAM images obtained during OHRhMOP + diz film formation; UV–vis and AFM characterization of the post-synthetic modification of MOP films; parameters used to simulate the Rh 3d high-resolution XPS spectra of MOP films; and comparison of the performance of MOP and PIM ultrathin films in CO_2_/N_2_ separation (PDF)
