# Supplementary material for: Influence of the Surface Chemistry of Metal–Organic Polyhedra in Their Assembly into Ultrathin Films for Gas Separation
Source: ACS Appl Mater Interfaces. 2022 Jun 3;14(23):27495–506. doi: 10.1021/acsami.2c06123 (PMC9204701; doi:10.1021/acsami.2c06123)
Supplement: Supplementary file 1 — am2c06123_si_001.pdf [file am2c06123_si_001.pdf]

Supporting information for the manuscript

**Influence of the surface chemistry of metal-organic polyhedra in their assembly into ultrathin films for gas separation**

Inés Tejedor[a,b], Miguel A. Andrés[a,b], Arnau Carné-Sánchez[c], Mónica Arjona[b], Marta Pérez-Miana[a,d], Javier Sánchez-Laínez[a,d], Joaquín Coronas[a,d], Philippe Fontaine[e], Michel Goldmann[e,f], Olivier Roubeau[a], Daniel Maspoch[c] and Ignacio Gascón[a,b*]

[a] Instituto de Nanociencia y Materiales de Aragón (INMA), CSIC and Universidad de Zaragoza, Zaragoza 50009, Spain.

[b] Departamento de Química Física, Universidad de Zaragoza, 50009 Zaragoza, Spain.

[c] Catalan Institute of Nanoscience and Nanotechnology (ICN2), CSIC and The Barcelona Institute of Science and Technology, Campus UAB, Bellaterra, 08193 Barcelona, Spain.

[d] Chemical and Environmental Engineering Department, Universidad de Zaragoza, 50018 Zaragoza, Spain.

[e] Synchrotron SOLEIL, L'Orme des Merisiers, Saint-Aubin, BP 48, 91192, Gif-sur-Yvette, France.

[f] Institut des NanoSciences de Paris, UMR 7588 CNRS, Sorbonne Université, 4 place Jussieu, 75252 Paris Cedex 05, France.

* Corresponding author. E-mail: igascon@unizar.es

**OHRhMOP alkyl functionalization with diz in solution**

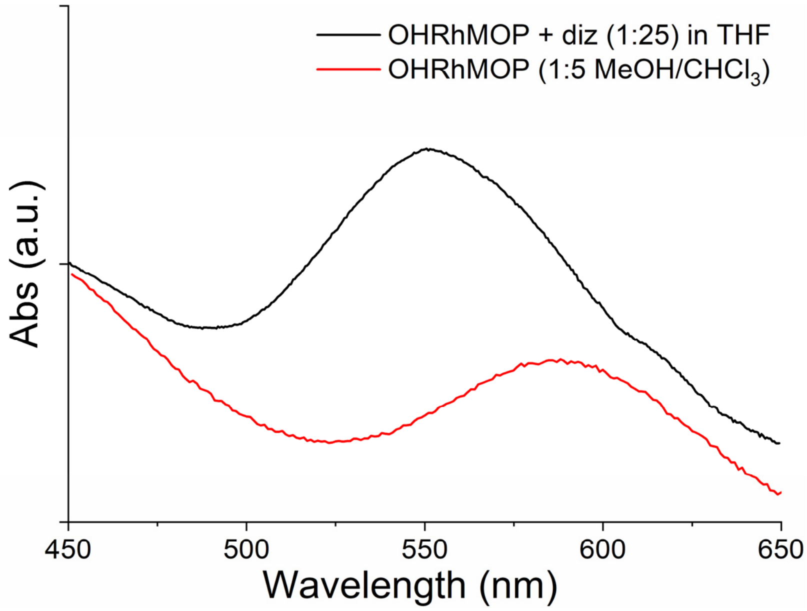


Figure S1. Absorption spectra (450-650 nm range) of OHRhMOP dissolved in methanol/chloroform (1:5) and the product formed after the addition of ca. $3.8\times10^{-3}$ mmol of diz to a dispersion of ca. $1.5\times10^{-4}$ mmol OHRhMOP in 2 mL of THF. The maximum absorption at ca. 552 nm after diz addition indicates that all the dirhodium paddlewheels of OHRhMOP are coordinated to one diz, obtaining OHRhMOP(diz)$_{12}$.

## *In situ* synchrotron characterization of Rh-MOP Langmuir films

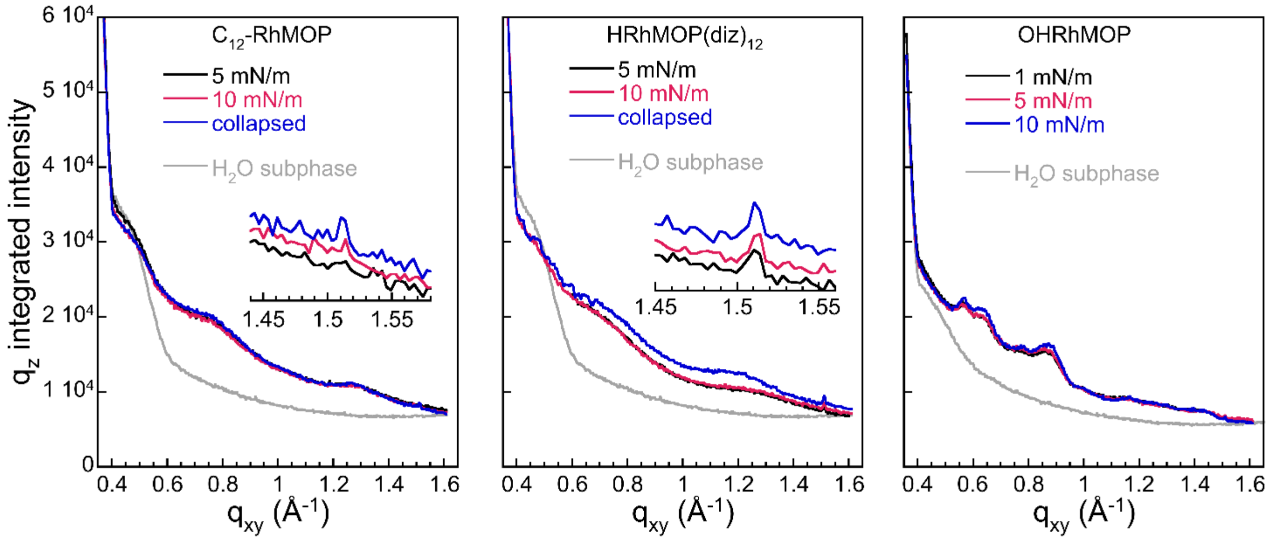


Figure S2. Raw GIXD data for $C_{12}$RhMOP (left), HRhMOP(diz)$_{12}$ (middle) and OHRhMOP (right), at the indicated pressures. The water subphase data are shown as grey lines. Insets highlight the q range exhibiting the Bragg peak of alkyl chains ordering, in the case of $C_{12}$RhMOP and HRhMOP(diz)$_{12}$.

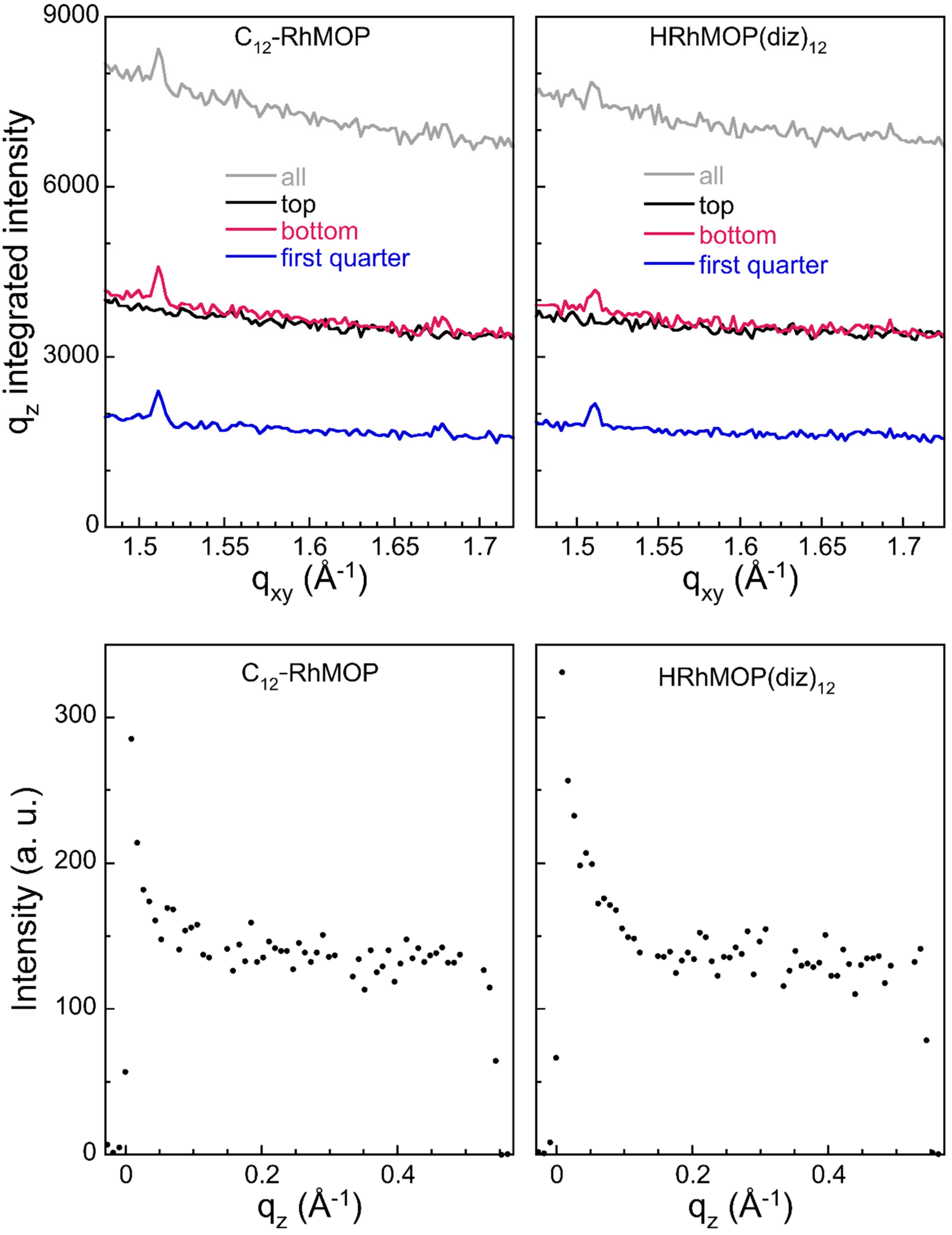


Figure S3. Top: high q portion of GIXD data for $C_{12}$RhMOP (left, collapsed) and HRhMOP(diz)$_{12}$ (right, 10 mN/m), integrated over only the bottom half, top half, bottom first quarter or the whole detector, as indicated. The Bragg peak at *ca.* 1.51 Å$^{-1}$ characteristic of alkyl chain interdigitation/order is not present in the data at higher $q_z$. Bottom: intensity of the alkyl chains Bragg rod *vs.* $q_z$, $C_{12}$RhMOP (left), HRhMOP(diz)$_{12}$ (right) at 10 mN/m.

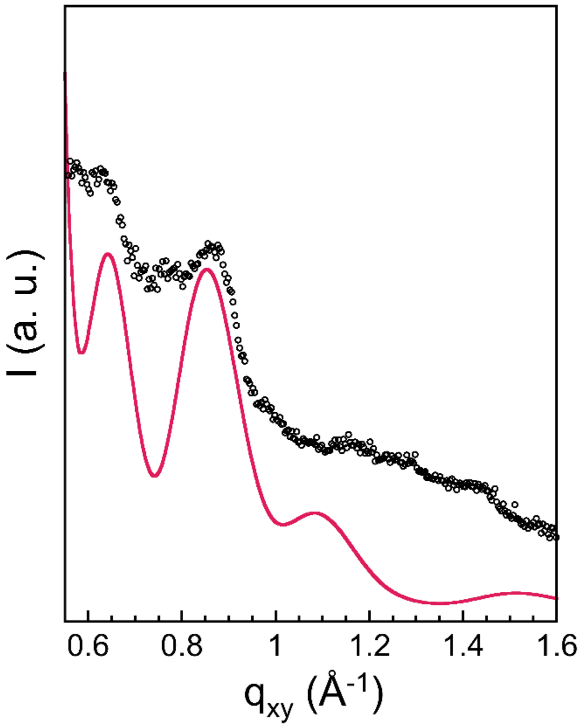


Figure S4. GIXD data for OHRhMOP at the gas-water interface at 10 mN/m, after correction for the water subphase. The red line is the diffusion form factor of core-shell spheres with an empty (SLD = 0) core of 5 Å radius and a dense shell of 11.5 Å thickness (SLD = $2x10^{-6}$ $Å^{-2}$), considering a pinhole instrumental smearing dQ/Q of 5 %, that can only account for the two stronger peaks at 0.63 and 0.87 $Å^{-1}$.

**Rh-MOP films deposition for gas separation studies**

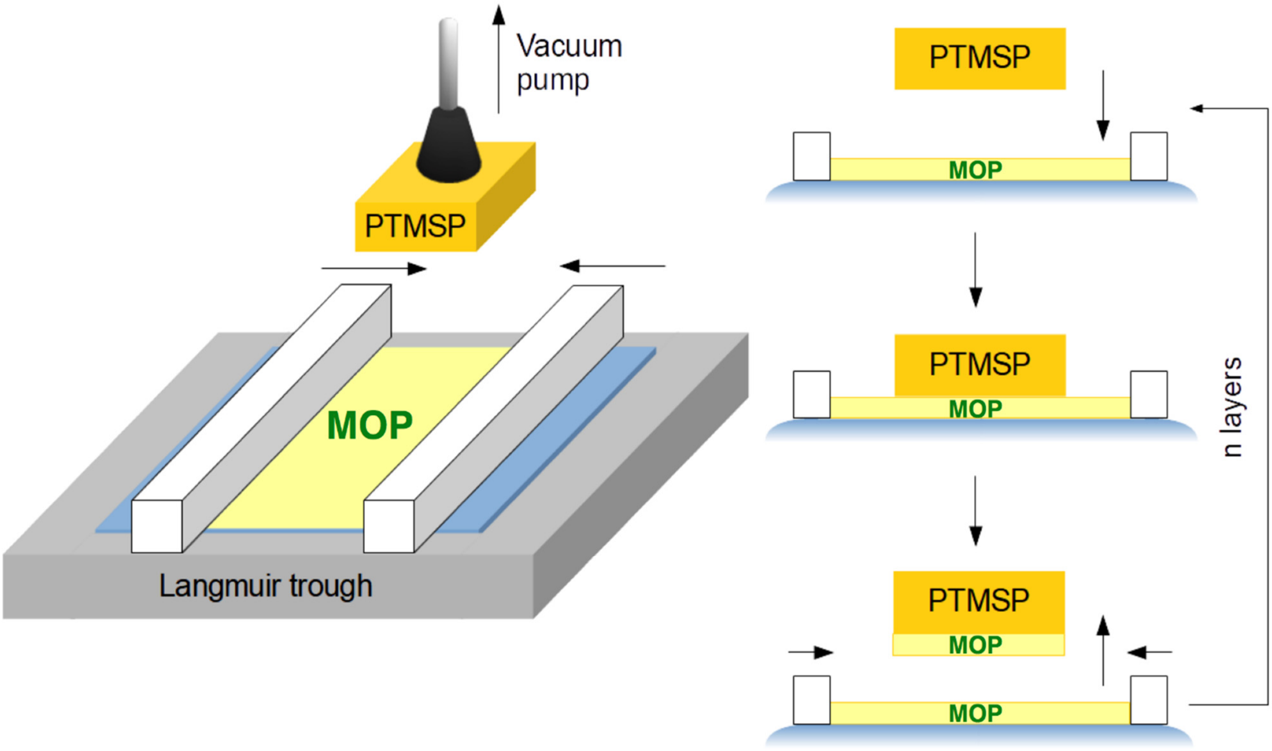


Scheme S1. LS sequential deposition of MOP monolayers onto PTMSP supports. One MOP monolayer is deposited each time that the support contacts the film formed at the air-liquid interface. After each transfer, the film is dried with $N_2$ at ambient temperature and the transference is repeated as many times as necessary to obtain films with the desired number of Rh-MOP monolayers.

## Rh-MOP films characterization

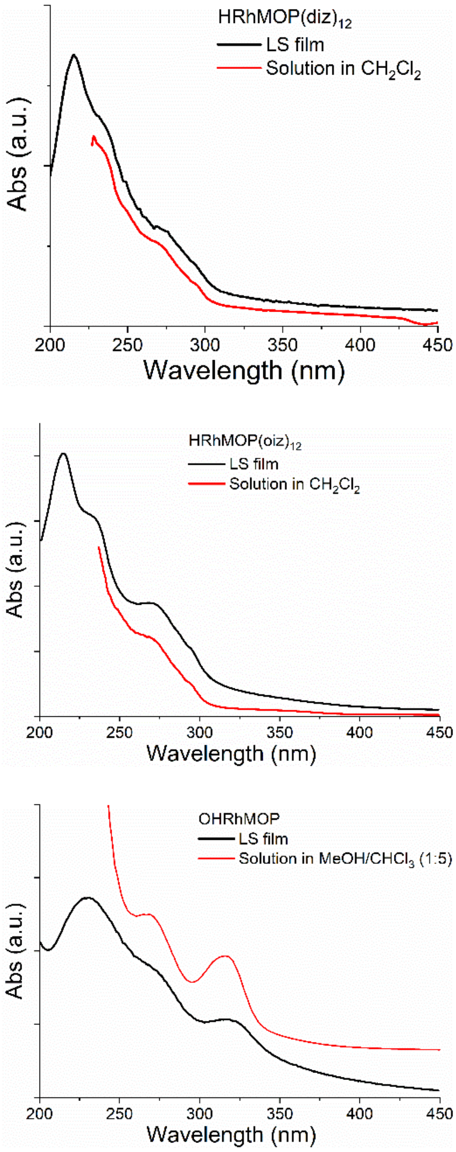


Figure S5. UV-Vis spectra for the three Rh-MOPs studied. Solution spectra and LS films deposited onto quartz substrates are compared for each Rh-MOP.

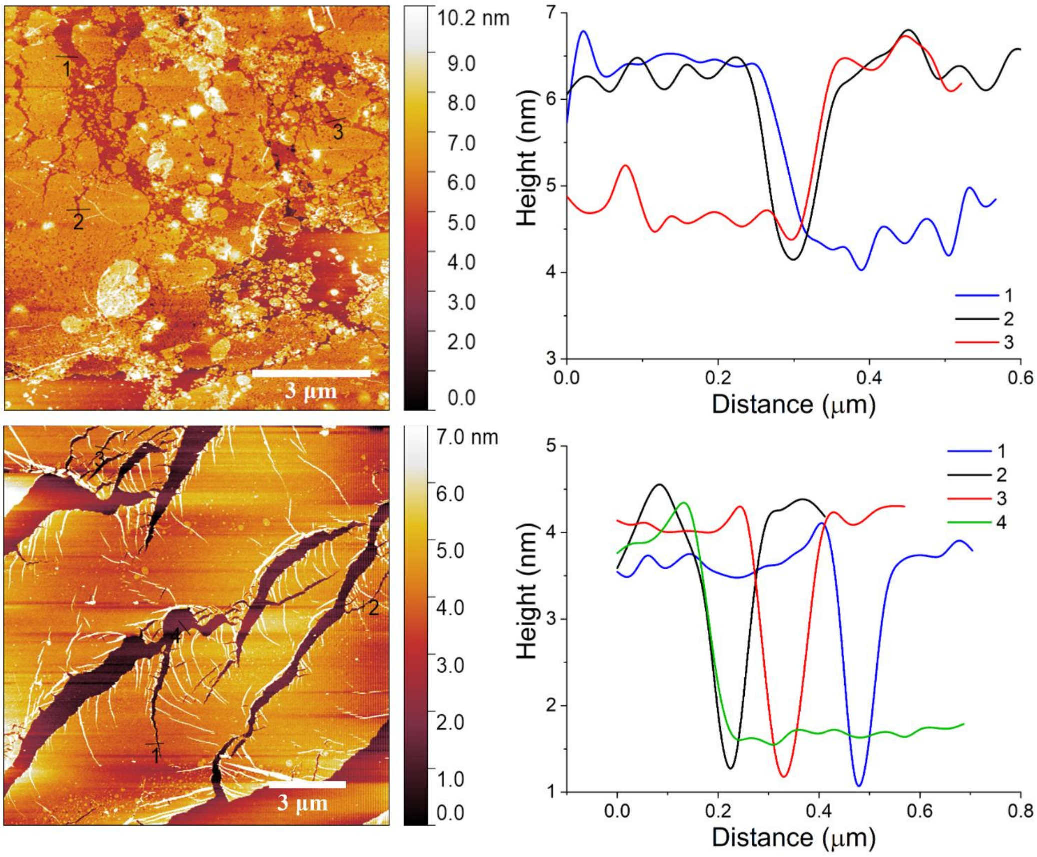


Figure S6. Representative AFM topography images from HRhMOP(oiz)$_{12}$ and HRhMOP(diz)$_{12}$ LS films transferred onto quartz substrates at 20 mN/m used to evaluate the film thickness.

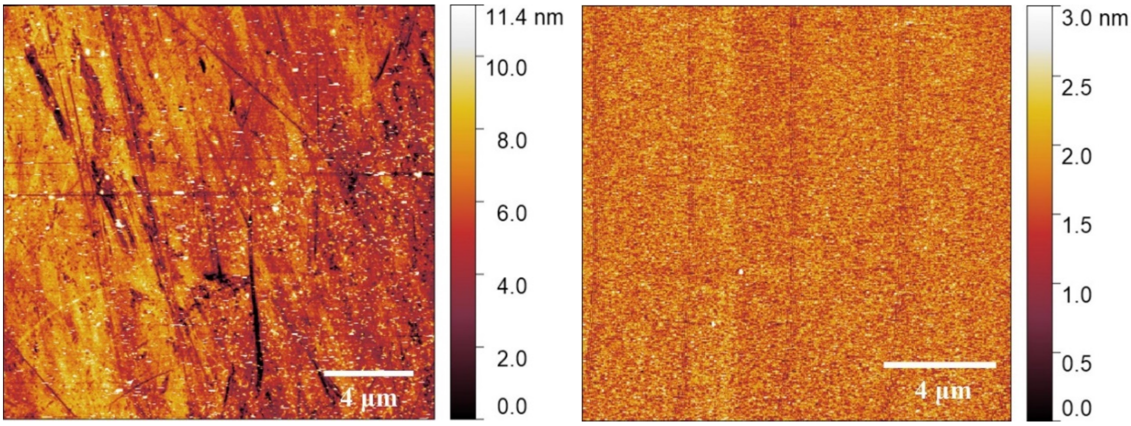


Figure S7. Representative AFM topography image of quartz, left, and a Si(100), right, substrates before MOP film deposition.

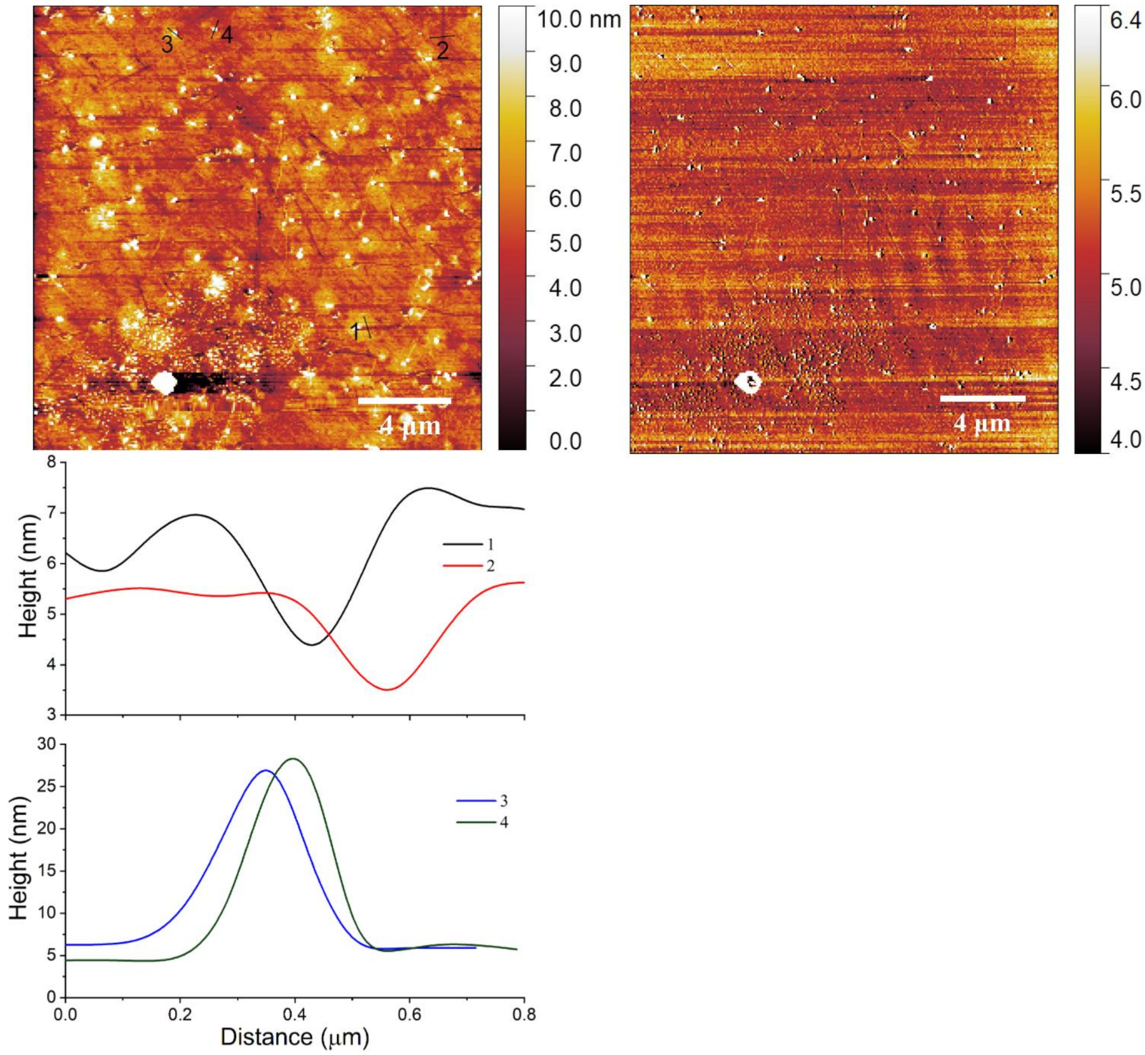


Figure S8. Representative AFM topography and phase images from a OHRhMOP LS film transferred at 2 mN/m and evaluation of film thickness and defects dimensions.

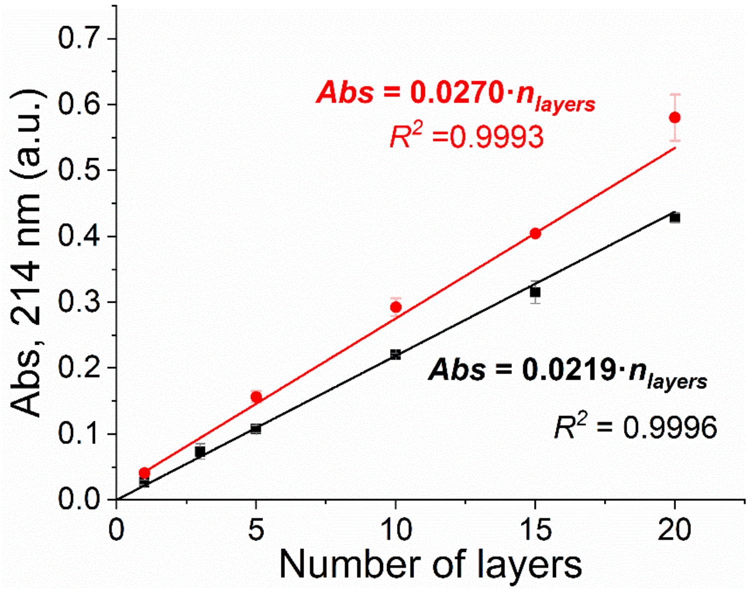


Figure S9. Linear increase of the absorbance at 214 nm vs. the number of Rh-MOP LS layers transferred at 20 mN/m onto quartz substrates (● HRhMOP(oiz)$_{12}$;■: HRhMOP(diz)$_{12}$)

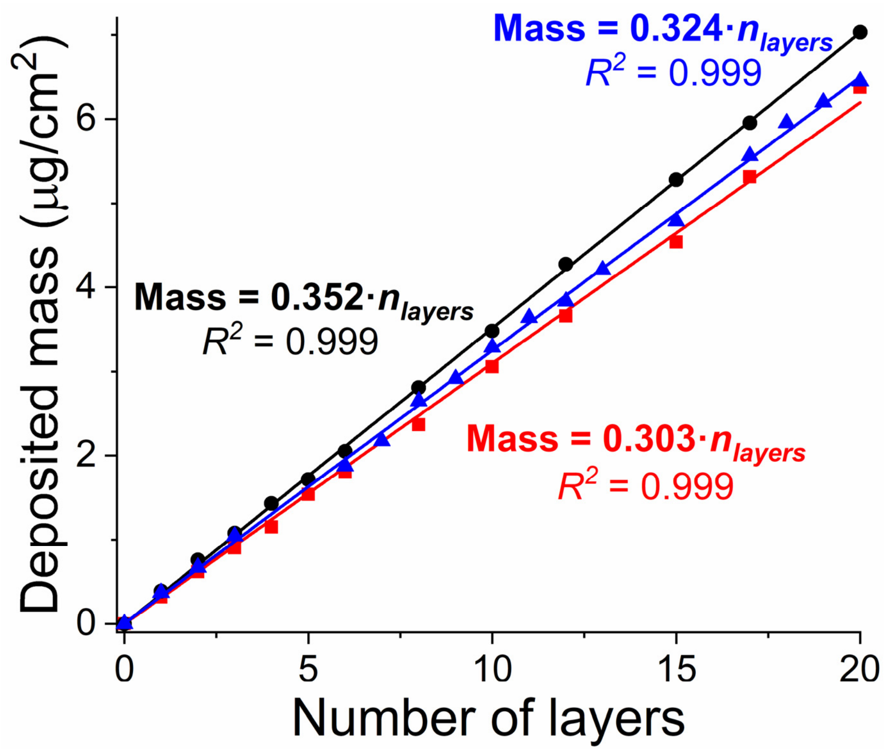


Figure S10. Rh-MOP mass deposited onto QCM disks at 20 mN/m versus the number of LS layers transferred (■: $C_{12}$RhMOP; ▲: HRhMOP(oiz)$_{12}$, ●: HRhMOP(diz)$_{12}$).

## Air-water interfacial formation of alkyl-functionalized Rh-MOP films

<table>
<tr><th>OHRhMOP / diz ratio</th><th colspan="3">BAM images</th></tr>
<tr><td>1:25</td>
<td>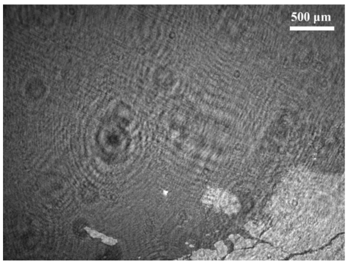<br>0.1 mN·m$^{-1}$<br>(5.0 nm$^2$/molecule)</td>
<td>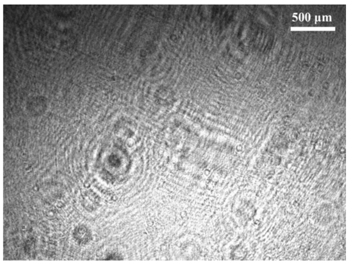<br>19.1 mN·m$^{-1}$<br>(2.6 nm$^2$/molecule)</td>
<td>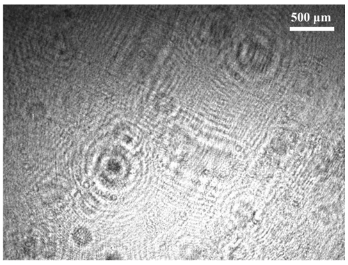<br>32.8 mN·m$^{-1}$<br>(1.8 nm$^2$/molecule)</td></tr>
<tr><td>1:50</td>
<td>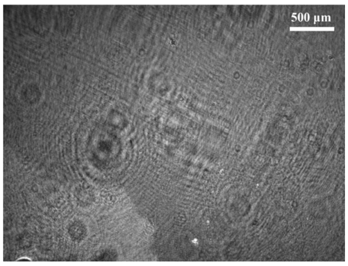<br>1.4 mN·m$^{-1}$<br>(6.0 nm$^2$/molecule)</td>
<td>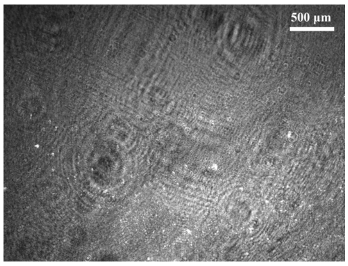<br>15.9 mN·m$^{-1}$<br>(4.0 nm$^2$/molecule)</td>
<td>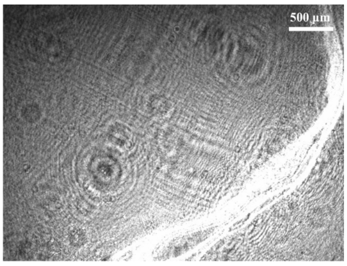<br>28.8 mN·m$^{-1}$<br>(3.3 nm$^2$/molecule)</td></tr>
</table>

Figure S11. Brewster Angle Microscope (BAM) images obtained during OHRhMOP + diz film compression at indicated surface pressures and the corresponding areas per molecule. OHRhMOP + diz different ratios were used in the experiments (1:25 in top images, and 1:50 in bottom images, respectively).

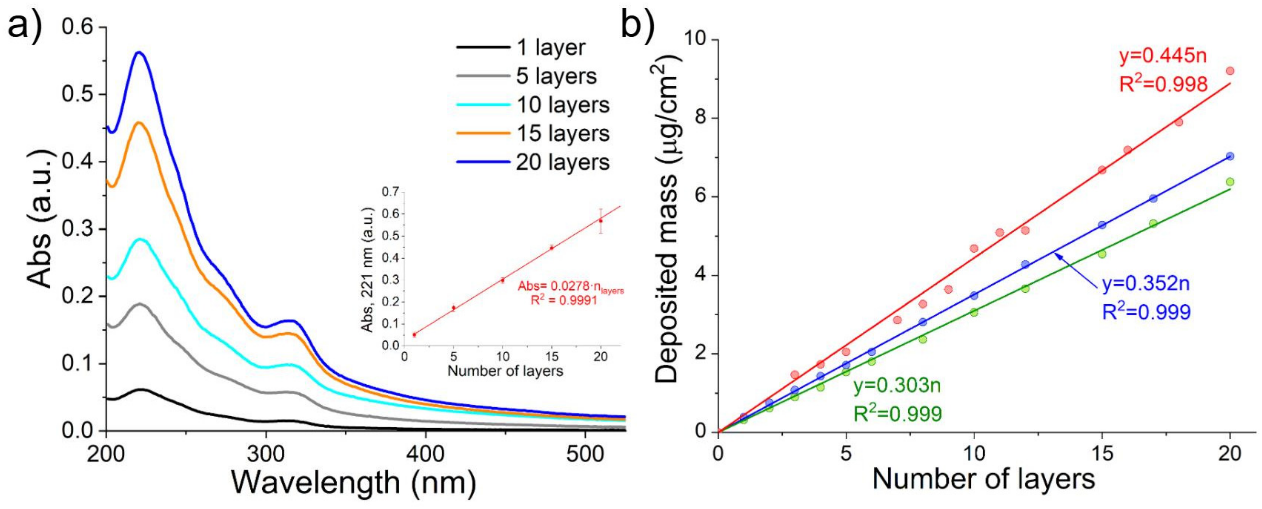


Figure S12. Characterization of the films obtained from OHRhMOP + diz (1:25) reaction at the air-liquid interface: a) UV-Vis spectra from sequential deposition of LS films transferred onto quartz at 20 mN/m. Inset: Linear increase of the absorbance at 221nm vs. the number of LS layers transferred. b) Mass deposited onto QCM disks vs. the number of LS layers transferred (red line: OHRhMOP + diz; blue line: HRhMOP(diz)$_{12}$, green line: C$_{12}$RhMOP).

**Post-synthetic modification of alkyl-functionalized Rh-MOP films**

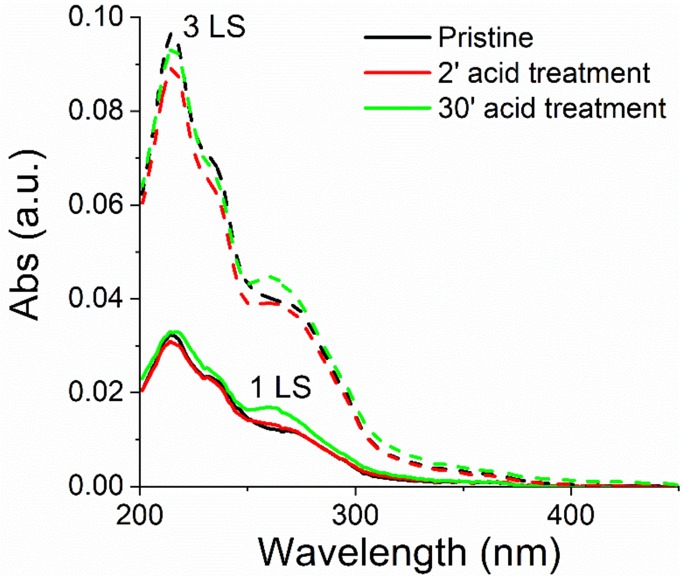


Figure S13: UV-Vis spectra from HRhMOP(diz)$_{12}$ LS films deposited onto quartz at 20 mN/m before and after the acid treatment: 1 layer (continuous line) and 3 layers (dashed line).

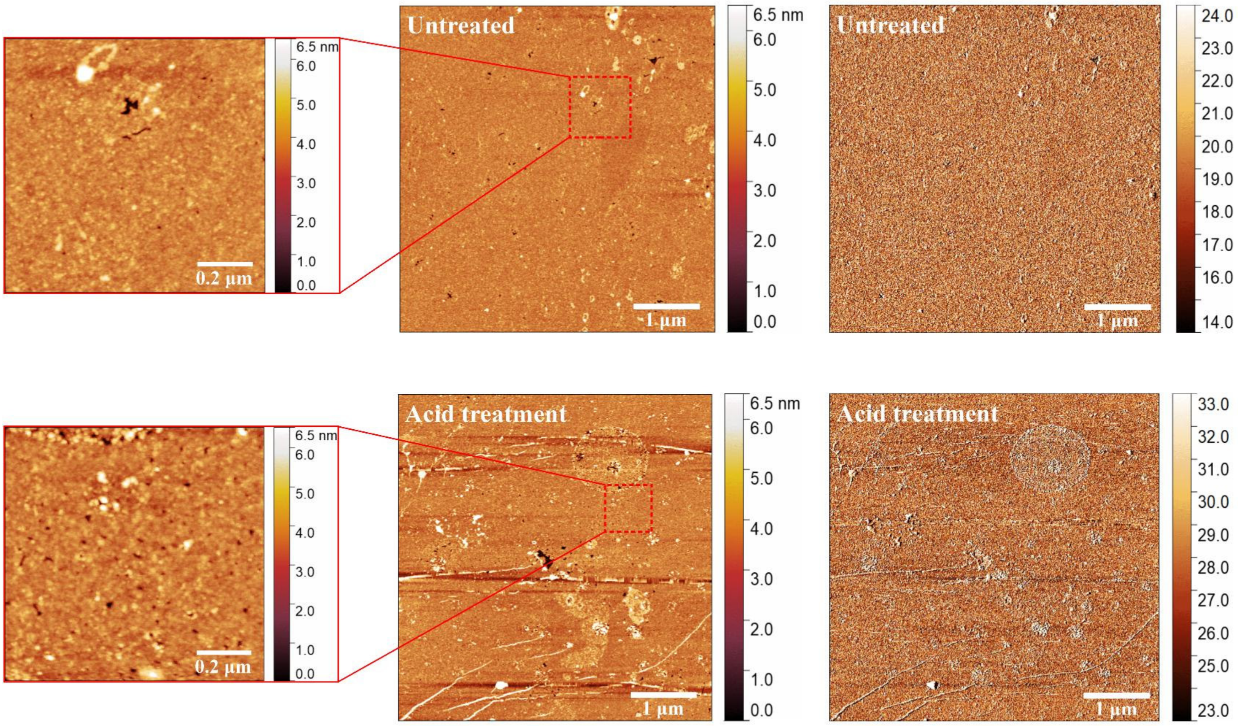


Figure S14. Representative AFM topography and phase images from a HRhMOP(diz)$_{12}$ LS film (1 layer) deposited onto Si (100) before and after acid treatment with HCl vapors.

Table S1: Parameters of the components used to simulate the Rh 3d high resolution XPS spectra (see Figure 9) of OHRhMOP (powder), 1 LS film deposited at 20 mN/m after OHRhMOP + diz (1:25) reaction at the air-liquid interface and drop-cast film obtained after OHRhMOP + diz (1:25) reaction in THF.

| **OHRhMOP + diz (1:25), 1LS film** | | | |
|---|---|---|---|
| **B.E. (eV)** | **FWHM** | **Area** | **%Area** |
| 310.21 | 1.457 | 4869 | 34.8 |
| 313.46 | 1.936 | 2012 | 14.3 |
| 308.76 | 1.316 | 3473 | 24.9 |
| 314.88 | 2.234 | 3641 | 25.9 |
| **OHRhMOP, powder** | | | |
| **B.E. (eV)** | **FWHM** | **Area** | **%Area** |
| 308.99 | 1.481 | 5073 | 60.7 |
| 313.80 | 2.121 | 3299 | 39.3 |
| **OHRhMOP + diz (1:25) in THF, drop-cast film** | | | |
| **B.E. (eV)** | **FWHM** | **Area** | **%Area** |
| 310.13 | 1.492 | 389 | 19.7 |
| 313.47 | 2.491 | 647 | 32.6 |
| 308.66 | 1.961 | 819 | 41.4 |
| 315.09 | 1.552 | 124 | 6.3 |

Table S2: Comparison of the performance of MOP and PIM ultrathin films (30 LS monolayers deposited onto PTMSP membranes) in $CO_2/N_2$ (10/90 in volume) separation at 35 ºC. At least 2 different samples were fabricated and measured to provide the corresponding error estimations.

| **LS selective layer** | **Feed pressure (bar)** | **$CO_2$ permeance (GPU)** | **$N_2$ permeance (GPU)** | **$CO_2/N_2$ selectivity** |
|---|---|---|---|---|
| PIM-EA-TB($H_2$)[1] | 1 | 118 ± 2 | 8.5 ± 0.2 | 13.8 ± 0.5 |
| | 3 | 114 ± 6 | 8.5 ± 0.4 | 13.5 ± 0.1 |
| PIM-TMN-Trip[2] | 1 | 134 ± 21 | 11.0 ± 1.7 | 12.1 ± 0.1 |
| | 3 | 129 ± 18 | 11.1 ± 1.5 | 11.6 ± 0.1 |
| $C_{12}$RhMOP[3] | 1 | 195 ± 26 | 19.5 ± 3.7 | 10.1 ± 0.6 |
| | 3 | 183 ± 24 | 19.7 ± 3.2 | 9.3 ± 0.3 |
| HRhMOP(diz)$_{12}$ | 1 | 118 ± 11 | 9.2 ± 0.4 | 12.8 ± 1.8 |
| | 3 | 100 ± 25 | 8.2 ± 0.7 | 12.1 ± 1.9 |
| OHRhMOP + diz | 1 | 212 ± 42 | 22.4 ± 5.0 | 9.5 ± 0.3 |
| | 3 | 209 ± 22 | 24.8 ± 0.3 | 8.4 ± 0.8 |

## REFERENCES

1. Benito, J.; Sanchez-Lainez, J.; Zornoza, B.; Martin, S.; Carta, M.; Malpass-Evans, R.; Tellez, C.; McKeown, N. B.; Coronas, J.; Gascon, I., Ultrathin Composite Polymeric Membranes for $CO_2/N_2$ Separation with Minimum Thickness and High $CO_2$ Permeance. *Chemsuschem* **2017,** *10* (20), 4014-4017

2. Benito, J.; Vidal, J.; Sanchez-Lainez, J.; Zornoza, B.; Tellez, C.; Martin, S.; Msayib, K. J.; Comesana-Gandara, B.; McKeown, N. B.; Coronas, J.; Gascon, I., The Fabrication of Ultrathin Films and their Gas Separation Performance from Polymers of Intrinsic Microporosity with Two-Dimensional (2D) and Three-Dimensional (3D) Chain Conformations. *Journal of Colloid and Interface Science* **2019,** *536*, 474-482.

3. Andres, M. A.; Carne-Sanchez, A.; Sanchez-Lainez, J.; Roubeau, O.; Coronas, J.; Maspoch, D.; Gascon, I., Ultrathin Films of Porous Metal-Organic Polyhedra for Gas Separation. *Chemistry-A European Journal* **2020,** *26* (1), 143-147.
